# Ancestral Protein‐Based Lighting

**DOI:** 10.1002/adma.202420303

**Published:** 2025-06-25

**Authors:** Stephanie Willeit, Alexander Mauz, David Gutiérrez‐Armayor, Joseph Arbash, Jesús Agustín Banda‐Vázquez, Sergio Martí, Pedro B. Coto, Rubén D. Costa

**Affiliations:** ^1^ Technical University of Munich Campus Straubing for Biotechnology and Sustainability Chair of Biogenic Functional Materials Schulgasse 22 94315 Straubing Germany; ^2^ Institute of Advanced Materials (INAM) Universitat Jaume I Av. Vicent Sos Baynat, s/n Castellón de la Plana 12071 Spain; ^3^ Nanomaterials and Nanotechnology Research Center (CINN) Spanish National Research Council (CSIC) and Donostia International Physics Center (DIPC) Avenida de la Vega 4–6 El Entrego 33940 Spain

**Keywords:** ancestral sequence reconstruction, bio‐hybrid light‐emitting diodes, fluorescent protein, protein‐lighting, photon manipulation

## Abstract

Protein‐optoelectronics is a paradigm toward eco‐designed and sustainable technologies. The challenge is, however, how to preserve the native activity of proteins upon device fabrication/operation in non‐native environments (solvents, organic/inorganic interfaces, and working temperatures/irradiations). Herein, a new vision to identify and design ancestral‐like fluorescent proteins (FPs) is proposed. Using ancestral sequence reconstruction (ASR) out of a large dataset (221) of the best modern FPs suitable for photon down‐conversion in bio‐hybrid light‐emitting diodes (Bio‐HLEDs) a historical‐genetic reconstruction (family tree) was obtained, identifying a possible common ancestral FP. This computationally designed protein is produced in bacteria, featuring outstanding photoluminescence quantum yields in solution (e.g., 90%/80% for green‐/red‐emitting forms) and a strong tendency to agglomerate in polymer coatings. This resulted in red‐emitting Bio‐HLEDs that outperformed the reference with ≈2‐fold enhanced stabilities. The resplendent green‐/red‐emission of ancestral‐like FP itself and its respective devices led us to coin this new protein as QuetzalFP. Overall, it is set in ASR as an effective concept to reshape protein‐optoelectronics allowing us to identify i) many interesting ancestral FPs for lighting and ii) QuetzalFP as stepping‐stone platform for protein engineering.

## Graphical abstract

The ancestral sequence reconstruction (ASR) method applied to a large dataset of modern fluorescent proteins (FPs) led to a bright ancestral‐like FP suitable for photon down‐conversion in bio‐hybrid light‐emitting diodes (Bio‐HLEDs).

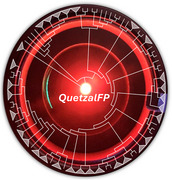

## 1 Introduction

The continuous adaptation of living organisms through evolution^[^ ^1^, ^2^ ^]^ has endowed proteins with a functional diversity finely programmed to succeed in the implementation of their biological tasks.^[^ ^3^, ^4^ ^]^ Using present‐day (extant) proteins as a starting point, synthetic biology has matured a toolbox to customize functionality for in vivo technologies, such as bioimaging, biosensing, bioelectronics, etc.^[^ ^5^, ^6^, ^7^ ^]^ In the emerging field of protein‐based materials for optoelectronics,^[^ ^8^, ^9^, ^10^, ^11^, ^12^ ^]^ protein stabilization is key to keep their biofunctionality in non‐native environments. Thus, an ongoing research effort aims to overcome the lack of resistance of extant proteins to irradiation and temperature stress, organic solvents, water‐free and/or water‐less environments, and unfriendly inorganic‐organic interfaces.

In this context, a leading example in lighting is the bio‐hybrid light‐emitting diode (Bio‐HLED),^[^ ^8^, ^11^, ^12^, ^13^ ^]^ in which either a coating of fluorescent proteins (FPs) embedded into polymers (mixture of branched and linear polyethylene oxides, polymethylmethacrylate, polyvinyl alcohol or hydroxypropyl cellulose), silica nanoparticles, and metal‐organic frameworks or dry protein‐films are placed onto the high‐energy emitting LEDs as photon down‐converting filters.^[^ ^13^, ^14^, ^15^, ^16^, ^17^, ^18^, ^19^, ^20^ ^]^ The aim is to replace the commercial filters based on rare earth and/or toxic emitters (inorganic phosphors) that are hampering the sustainability transition of the lighting sector.^[^ ^21^ ^]^ Indeed, the interest in extant FPs in lighting is fueled by i) their high photoluminescence quantum yields (*ϕ*) with narrow emissions covering the whole visible range and ii) their ecological advantages, such as unlimited low‐cost production using bacteria, whole decomposable nature, and designability to be easily encoded into their sequences.^[^ ^8^, ^9^, ^10^, ^11^, ^12^, ^13^, ^14^, ^15^, ^16^, ^17^, ^18^, ^19^, ^20^ ^]^ However, the host matrix/application environment is radically different from the natural cellular environment of FPs, resulting in structural stress that limits the device performance.^[^ ^14^, ^16^, ^19^, ^20^, ^22^, ^23^, ^24^, ^25^, ^26^, ^27^ ^]^ This also includes the working temperatures that typically range from 35 to 80 °C upon continuous excitation of the FP‐based color filters and lead to a temperature‐induced emission quenching and FP denaturation (ionic‐to‐neutral chromophore deactivation, cis‐trans isomerization, and oxydation).^[^ ^22^, ^23^ ^]^ To date, protein stabilization concepts related to i) enhanced matrices (oxygen/moisture barriers and fine‐tuned FP‐polymer interfaces),^[^ ^14^, ^18^, ^23^, ^25^, ^26^ ^]^ ii) rational design of genetically encoded structural features^[^ ^19^, ^24^ ^]^ and iii) post‐translational chemical functionalization of modern FPs^[^ ^15^, ^17^, ^20^, ^27^ ^]^ have successfully been implemented. In addition, protein‐based materials for other technological fields have also been optimized via structural rationalization,^[^ ^28^, ^29^ ^]^ mutant libraries combinations^[^ ^30^ ^]^ and directed evolution.^[^ ^31^, ^32^, ^33^ ^]^

Herein, we propose a radically new vision for protein stabilization: why not to rebuild ancestral‐like FPs for the future of lighting technologies such as Bio‐HLEDs? We hypothesized that rather than starting from extant FPs, which are adapted to conditions far milder than those needed for optoelectronic applications, we could use their possible ancestors as a starting point. Indeed, the origin of bioluminescence in the *Cnidaria* (jellyfish, corals, sea anemones, etc.) has recently been estimated to occur 540 million years ago.^[^ ^34^ ^]^ Our premises are that ancestral proteins *i*) were robust enough to allow functional diversification through mutational tolerance over generations^[^ ^35^, ^36^, ^37^, ^38^, ^39^ ^]^ and *ii*) were adapted to possibly harsher environmental conditions as the planet atmosphere was different eons ago.^[^ ^40^, ^41^, ^42^, ^43^, ^44^, ^45^, ^46^, ^47^, ^48^, ^49^ ^]^

To this end, we capitalize on biological studies trying to understand the evolutionary path that led to natural proteins in extant organisms from those extinct by means of ancestral sequence reconstruction (ASR).^[^ ^44^, ^50^ ^]^ These studies frequently report resurrected possible ancestral proteins (usually referred as ancestors or Anc,^[^ ^30^, ^44^, ^50^ ^]^ due to the ASR technique used to estimate such ancestral‐like protein sequences rather than historically factual ancestry) with increased stability and substrate promiscuity compared to their extant counterparts, yet this has not been applied to FPs in the field of optoelectronics. More specifically, ASR applied to FPs includes a handful number of works focused on the natural evolution of the color emission of FPs with less than 25 extant sequences,^[^ ^30^, ^51^, ^52^, ^53^ ^]^ concluding that green emission evolved independently several times under the lineage of FPs leading to other color emissions.

In this work, we have carried out ASR with an assembled database of 221 FP sequences that are found upon their satisfaction to a sequence alignment of 22 FPs that exhibit one or more desired properties (color emission, high *ϕ*, etc.) for Bio‐HLEDs. This allowed us to determine the last common ancestral‐like FP purposely biased for enhanced lighting properties rather than obtaining an accurate historical‐genetic reconstruction (FP family tree or phylogeny). Indeed, the reconstructed ancestral‐like FP featured i) heterologous production yield in *Escherichia coli* (*E. coli;* 7 mg L^−1^), ii) green‐ and red‐emitting forms, showing outstanding *ϕ* in solution (90% for emission at 506 nm and 79% for emission at 581 nm), iii) excellent resilience in polymer coatings, fairly keeping the main photoluminescence and thermal figures, and iv) red‐emitting Bio‐HLEDs that outperformed those with reference protein with ≈2‐fold enhanced stability. Since this reconstructed ancestral‐like FP and its respective devices exhibit resplendent green and red emissions, we coined this new ancestral‐like protein as QuetzalFP, after the bird that inhabits some tropical regions of Mexico and Central America and is etymologically related to the god of light Quetzalcoatl in Aztec mythology. The good performance in solution and polymer‐coatings of the QuetzalFP has been tentatively attributed to the synergistic effect of a tighter chromophore cavity with a simple H‐bonding network and a strong dimer structure that might promote agglomeration, as suggested by spectroscopy and thermocycling assays as well as quantum mechanics/molecular mechanics/molecular dynamics (QM/MM/MD) simulations.

In light of our findings, this work sets in ASR as a powerful concept to discover and to design proteins that could be more suitable for optoelectronics disclosing i) a large variety of extinct‐like FP relatives of high interest for lighting, optics and photonics and ii) the first stepping‐stone with QuetzalFP as an attractive platform for protein engineering.

## 2 Results and Discussion

**Figure 1:**
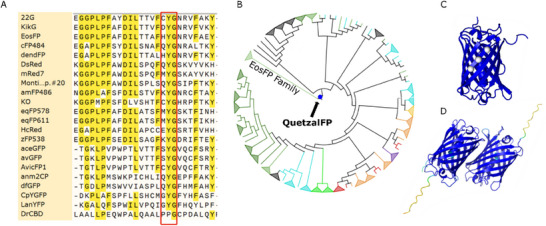
Ancestral sequence reconstruction of FPs. A) Zoom–in to the chromophore coding area of the alignment of the 22 seed sequences. B) Phylogenetic tree of the 221 sequences for ASR. Colors (either for leaves or for collapsed clades) are according to what is reported in the literature for the best Basic Local Alignment Search Tool (BLAST on https://blast.ncbi.nlm.nih.gov) hit for each of the initial extant FP sequences. In this tree, the closest node to the root is QuetzalFP (highlighted as a blue circle), the apparent last common ancestor of this entire phylogeny as well as the closest extant relative EosFP (shown in light green) to the root. C) AlphaFold2 model of the QuetzalFP monomer, highlighting the key glycine residues as white spheres.^[^ ^61^ ^]^ D) Dimeric model of the QuetzalFP showing a certainty > 90% in most of the structure (blue coloring). The chromophore region exhibits a certainty of ≈85%, as AlphaFold2 only models canonical amino acids based on the primary sequence.^[^ ^64^, ^67^ ^]^ The N‐ and C‐termini with the 6x‐HisTag at the former present lower confidence, which is usual in most modelled proteins.^[^ ^64^ ^]^

To reconstruct the ancestor‐like of extant β‐barrel FPs, we began by collecting the whole amino acid sequences from 22 representatives, such as cyan *Clavularia* cFP484,^[^ ^55^ ^]^ *Aequorea victoria* green‐fluorescent protein avGFP,^[^ ^56^ ^]^ yellow *Branchiostoma lanceolatum* LanYFP,^[^ ^57^ ^]^ *Fungia concinna* Kusabira‐Orange KO^[^ ^58^ ^]^ and *Discosoma* RED DsRED,^[^ ^55^ ^]^ as examples covering the whole visible emission range (see **Figure** **1**A; Figure S1, Supporting Information for details). After making a multiple sequence alignment (MSA) considering these sequences in their full length, we created a hidden Markov model to be used as a sequence profile for the search of homologs of naturally occurring and synthetic/engineered FPs that ultimately resulted in an extended dataset of 221 non‐identical FP members with sequences between 200 and 227 residues long, after filtering for sequence length and sequence identity redundancy (see Figure S1, Supporting Information for details). We then performed a new MSA on these 221 homologs to calculate the evolutionary relationships (phylogeny) among them and the ancestral sequences at the common ancestors (nodes). Node 222 of the ASR represents the last common ancestral‐like FP of the entire phylogeny, that is, QuetzalFP (Figure 1; Figure S1A, Supporting Information). As a control, publicly available synthetic/engineered FP sequences were eliminated in the phylogeny without affecting the QuetzalFP sequence, suggesting that human‐engineered FPs were not decisive in the reconstruction of the last common ancestral‐like FP (Figure S1B, Supporting Information). In addition, we found that the sequence of QuetzalFP is not identical to any other reported FP sequences, including natural, engineered and metagenome‐translated proteins in public sequence databases. Finally, AlphaFold2 modeling of QuetzalFP (Figure 1C)^[^ ^59^ ^]^ predicts a β‐barrel FP with most of the sequence structurally modelled with over 90% confidence. Furthermore, these results suggest that the likelihood of proper folding upon expression of QuetzalFP could be high, as the key glycine residues^[^ ^60^ ^]^ present in all known FP crystal structures are conserved with the lowest root mean square deviation (RMSD) to those in known crystal structures of FPs. Altogether, these findings encouraged us to work on the protein production of QuetzalFP.

**Figure 2:**
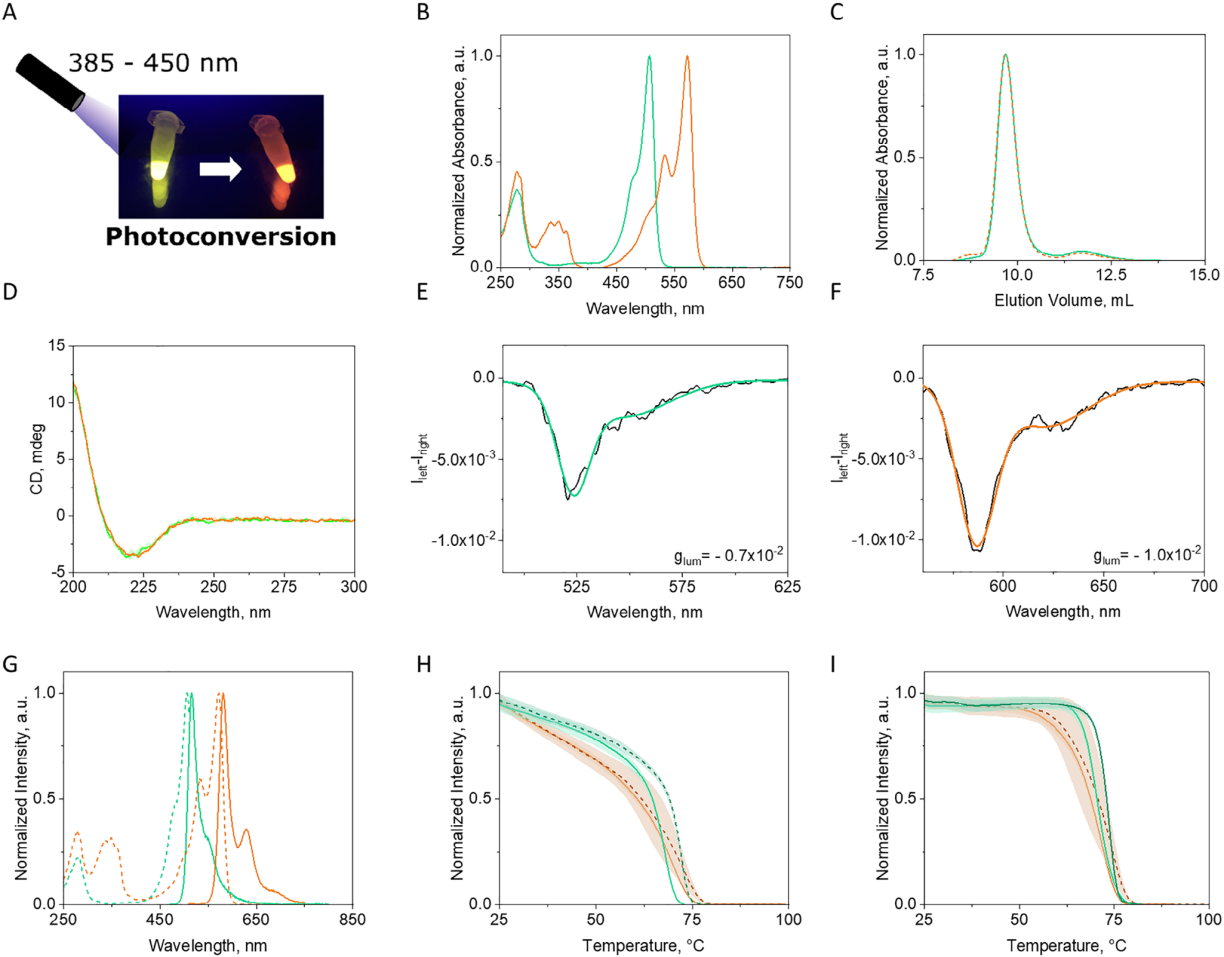
Characterization of QuetzalFP and EosFP(reference) in solution. Picture under UV light A), the UV–Vis absorption spectra B), SEC plot C), UV‐CD spectra D), CPL (E/F; black is CPL signal and color lines are the fittings) spectra, excitation (dashed line) and emission (solid line) spectra G) in solution of the green‐ and red‐emitting species of QuetzalFP (color code is adjusted to the respective protein species). Temperature melting H) and non‐reversible temperature plots I) of green‐ and red‐emitting species of QuetzalFP (solid line) and EosFP (dashed line). The shaded lines represent the deviation of 5 samples.

The heterologous expression of the synthesized QuetzalFP gene in *E. coli* gives rise to a resplendent green fluorescent protein (**Figure** **2**A,B). The green emission is in line with previous phylogenetic studies and ASR on FPs, suggesting that green is the primordial color emission as the chromophore formation involves fewer autocatalytic reactions than those of other colors.^[^ ^52^, ^61^ ^]^ QuetzalFP was purified with an average yield of 7 mg L^−1^, while analytical size exclusion chromatography (SEC, Figure 2C) showed that QuetzalFP exhibits a predominantly dimeric population with a molecular weight of 59 ± 0.5 kDa. UV‐CD absorption spectroscopy further confirmed the correct folding of the protein in the form of β‐barrel (Figure 2D), while the dissymmetry factor of the emission (*g*_lum_, −7 × 10^−3^) is also in line with those of other FPs, suggesting that the chromophore is well‐matured (Figure 2E).^[^ ^62^ ^]^ Indeed, the QuetzalFP dimer shows a well‐structured green fluorescence spectrum centered at 516 nm with a shoulder at 550 nm both with excited state lifetime (*τ*) values of 3.6 ns (**Table** **1**).

**Table 1:** Photophysical and thermal features of green‐ and red‐emitting species of QuetzalFP and EosFP in solution and polymer coatings.

Protein	Green species						Red species					
	*λ*_ex_/*λ*_em_ ^a)^ [nm]	*ϕ* ^b^ ^)^ [%]	*T*_m_/*T*_nr_ ^c^ ^)^ [°C]	*F_r_* ^d^ ^)^	*τ* ^e^ ^)^ [ns]	*ε* ^f^ ^)^ [M^−1^cm^−1^]	*λ*_ex_/*λ*_em_ ^a^ ^)^ [nm]	*ϕ* ^b^ ^)^ [%]	*T*_m_/*T*_nr_ ^c^ ^)^ [°C]	*F_r_* ^d^ ^)^	*τ* ^e^ ^)^ [ns]	*ε* ^f^ ^)^ [M^−1^cm^−1^]
QuetzalFP (solution)	506/516	90	62/71	43.1	3.6	104 300	571/581	79	61/69	40.3	4.5	95 300
EosFP (solution)	506/516	90^g)^	66/73	45.3	3.8	121 000	571/581	79^g)^	62/71	42.4	4.3	95 000
QuetzalFP (coating)	512/527	80	50/60	37.4	5.2	–	584/(599/ 628)	54	50/61	35.7	4.9	–
EosFP (coating)	512/527	77	51/63	38.7	4.6	–	585/(599/629)	59	55/67	40.9	4.5	–

^a)^ Excitation and emission wavelengths;

^b)^ Photoluminescence quantum yields;

^c)^ Melting temperature/non‐reversible temperature;

^d)^ Folding reversibility factor;

^e)^ Excited state lifetime at 450 nm;

^f)^ Molar extinction coefficient calculated according to Ward;^[^ ^66^ ^]^

^g)^ For the EosFP photoluminescence quantum yields in solution reported in the literature, the values vary depending on the methods/instruments used.^[^ ^54^, ^67^, ^68^ ^]^

Interestingly, the chromophore of QuetzalFP is not formed by the same amino acids as in archetypal green‐emitting FPs, such as wild‐type GFP or enhanced GFP (eGFP) that include either serine, tyrosine, and glycine (SYG) or threonine, tyrosine and glycine (TYG), respectively. Instead, QuetzalFP harbors a chromophore composed of histidine, tyrosine and glycine (HYG), which is known to be present in photoconvertible FPs.^[^ ^55^, ^63^, ^64^ ^]^ Indeed, exposure to irradiation at 380 nm in solution resulted in a color change from green to red (Figure 2A,B; Figure S2, Supporting Information). Upon full photoconversion, the red QuetzalFP species conserved the dimeric signature as indicated by SEC analysis (Figure 2C) and featured the same UV‐CD absorption spectrum and an increased *g*_lum_ value compared to that of the green form (Figure 2D,F). These findings confirm that the β‐barrel structure remains upon photoconversion and that the chromophore cavity changes enable an effective radiative deactivation. This is corroborated by the perfect match between the excitation and absorption spectra of the red QuetzalFP (Figure 2B,G).

Since photoconversion has been reported for other FPs with HYG chromophore,^[^ ^55^, ^63^, ^64^ ^]^ it is not surprising that its closest extant protein relative to QuetzalFP (EosFP; Figure 1; Figure S1, Supporting Information for details)^[^ ^54^ ^]^ that shares 88% of its sequence by global pairwise alignment (see Figure S2, Supporting Information for details) exhibits similar photophysical and thermal behaviors (Table 1, Figure 2H,I; Figures S3–S9, Supporting Information). On one hand, green QuetzalFP/EosFP species showed fluorescence spectra centered at 516 nm that quickly photoconverts to a red‐emitting species with an emission located at 581 nm, keeping similar photoluminescence features(Table 1, Figure 2G; Figures S3–S9, Supporting Information). On the other hand, both green and red species of QuetzalFP and EosFP also exhibited similar thermal figures in solution with a melting temperature (*T*_m_) ≈62 °C, non‐reversible temperature (*T*_nr_) of ≈70 °C, and a refolding capability (*F_r_*) of ≈40 (Figure 2H,I and Table 1).

**Figure 3:**
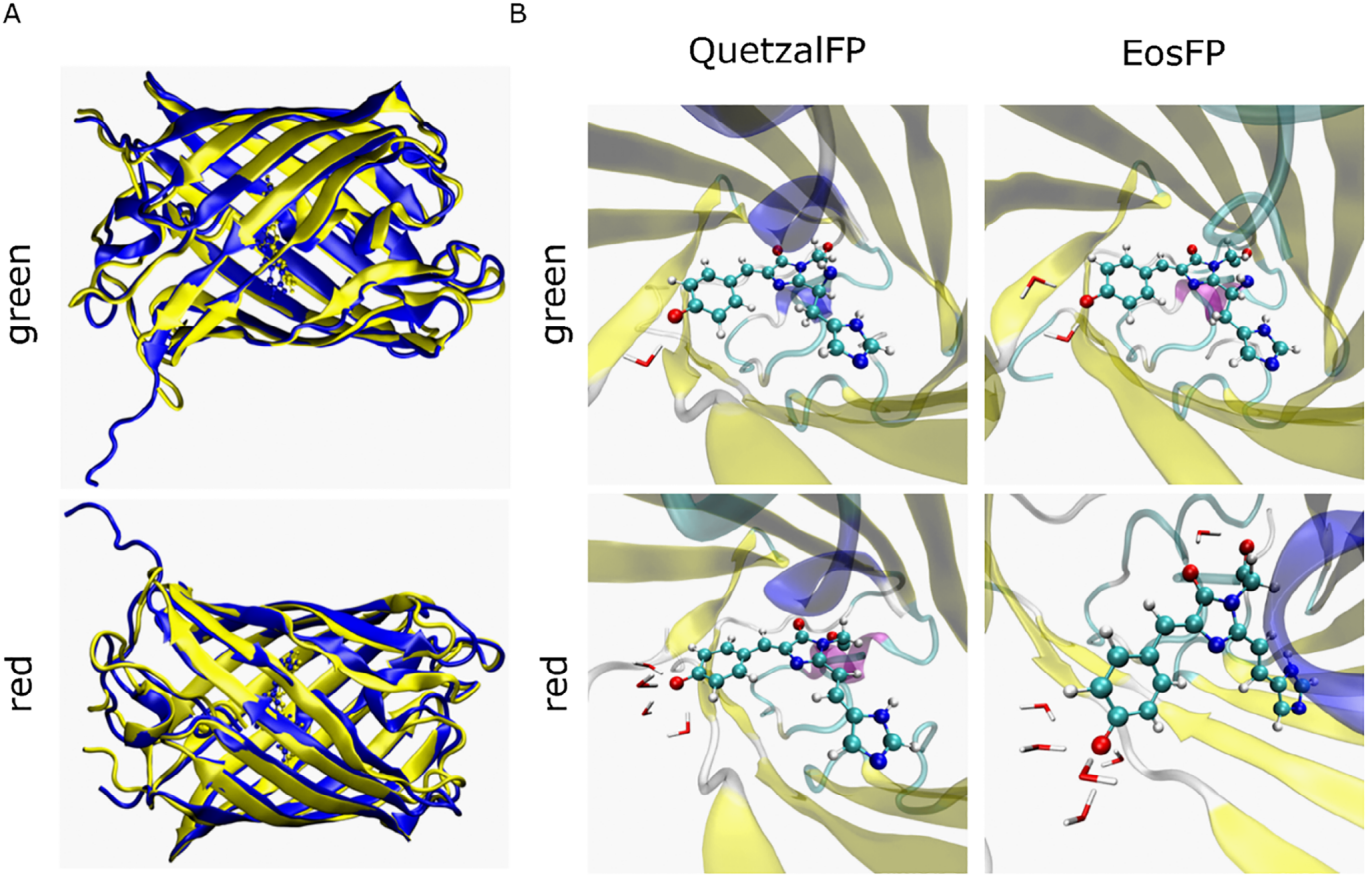
A) Superposition of QuetzalFP (blue) and EosFP (yellow) monomers for the green (top) and red (bottom) form of the chromophore in molecular dynamics simulations. B) Representative snapshots of the hydrogen bond interactions between the phenolic oxygen moiety of the chromophore and the water molecules of the solvent of the green (top) and red (bottom) QuetzalFP and EosFP (from left to right).

To further elucidate the role of structural and/or environmental factors on the photophysics of QuetzalFP and EosFP, we have carried out QM/MM/MD simulations for both systems in their dimeric form (see Discussion S1, Supporting Information for details). In general, both FPs exhibit similar β‐barrel structures with comparable main interactions between the residues of the chromophore binding pocket (see Tables S1 and S2, Figures S10–S20, Supporting Information for details). In addition, the chromophore exhibits similar degrees of torsion over the simulation time explored for the dihedral angles P (−13 ± 10° and −10 ± 12° in green form, while −5 ± 11° and −3 ± 11° in red form, respectively, Figure S15, Supporting Information) and I (−4 ± 10° and −2 ± 10° in green form and −2 ± 10° and 1 ± 9° in red form, respectively, Figure S16, Supporting Information). This is not surprising, since both proteins share a high degree of similarity in their amino acid sequences (vide supra). However, a more detailed analysis reveals three important differences. First, the number of binding interactions between the monomers in their green and red species is higher in QuetzalFP than that of EosFP (Figures S17–S20, Supporting Information), accounting for up to 4‐fold increased stabilization energy of the dimer with respect to the monomer forms (−597 versus −111 kcal mol^−1^ and −525 versus −213 kcal mol^−1^ for the green and red species of QuetzalFP versus EosFP, respectively). Second, the green species of QuetzalFP and EosFP show a similar volume of the chromophore binding pocket (575 ± 43 Å^3^ and 574 ± 37 Å^3^, respectively, Figures S18–S20, Supporting Information), while the corresponding red species differ, with QuetzalFP exhibiting a tendency toward a larger contraction of the chromophore binding pocket (516 ± 41 Å^3^) than its EosFP analog (552 ± 38 Å^3^). Finally, the H‐bond pattern of the water molecules in the chromophore binding pocket differs (see Table S3, Supporting Information for details). Specifically, the green species of QuetzalFP and EosFP show the same H‐bond interactions between the carbonyl group of the imidazole moiety and surrounding water molecules. By contrast, the phenolic moiety of the QuetzalFP bears a much simpler H‐bond interaction with just one water molecule, while an interplay with two water molecules is present in EosFP. In the red species, a more complex H‐bond interaction pattern is noted. Here, the carbonyl group of the imidazole moiety in EosFP interacts with one water molecule, while that of QuetzalFP does not show sizable interactions (Figure 3B). However, the phenolic moiety interacts with at least four water molecules in both FPs (Figure 3B; see Table S3, Supporting Information for details).

In light of the above mentioned, QuetzalFP shows i) a larger stabilization energy related to the dimeric form, ii) a smaller chromophore binding pocket, and iii) a simpler H‐bonding network around the chromophore (phenolic and imidazole moieties). Altogether, this suggests a highly emissive character.^[^ ^65^ ^]^ However, further theoretical and ultra‐fast transient spectroscopy studies analyzing the excited state dynamics of these systems are needed to disentangle the specific roles of each of these factors in both, radiative and non‐radiative deactivation processes.

**Figure 4:**
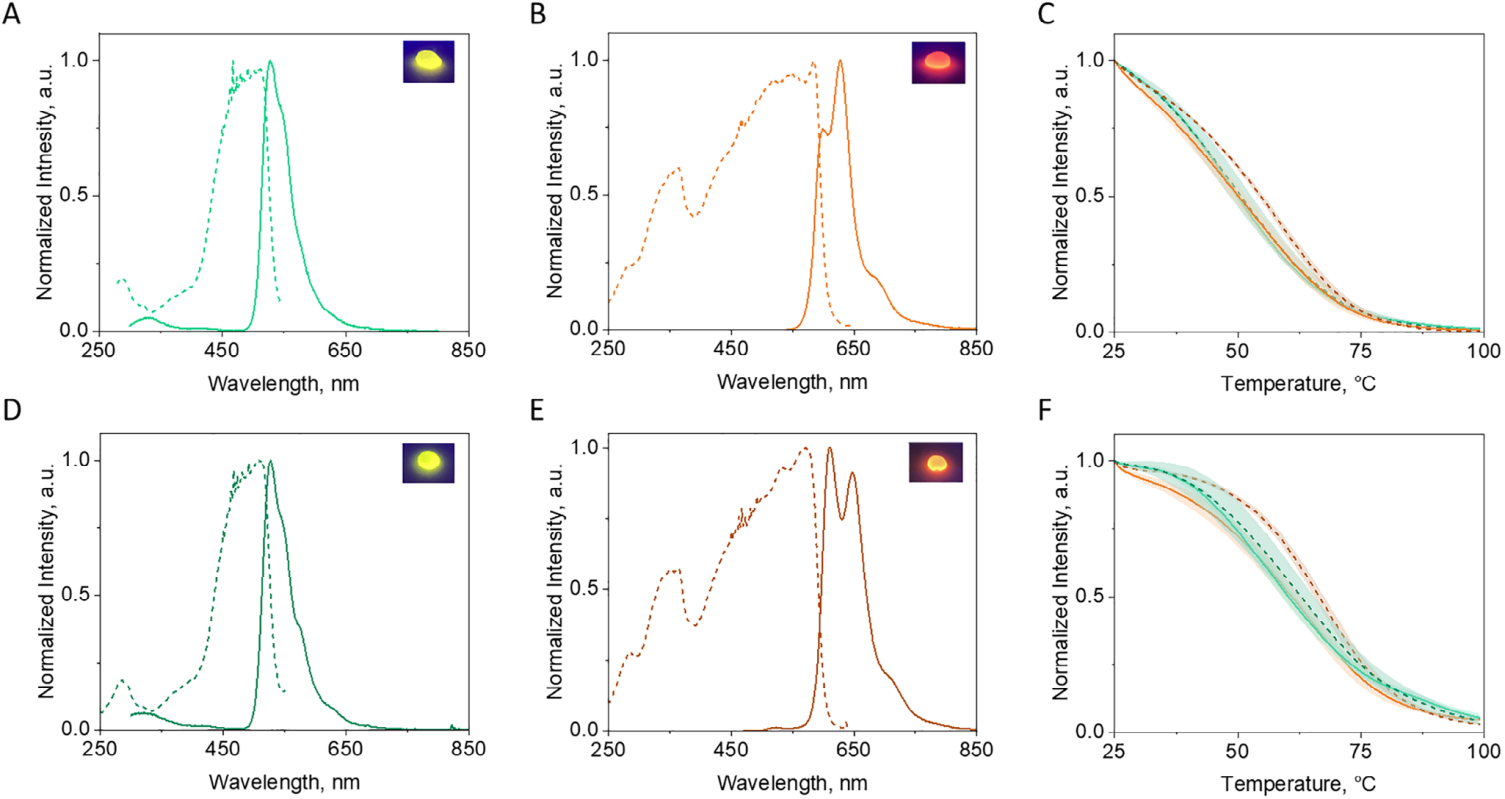
Characterization of green and red QuetzalFP (top) and EosFP (bottom) species in polymer coatings. Excitation (dashed line) and emission (solid line) spectra of green A) and red B) species of QuetzalFP and D) and E) for EosFP coatings (color code is adjusted to the respective protein species). Inset is the picture of the coatings under ambient and UV irradiation. Temperature melting C) and non‐reversible temperature F) plots of the green and red QuetzalFP (solid line) and EosFP (dashed line) coatings. The shaded lines represent the deviation of 5 samples.

Overall, the photoluminescent features of QuetzalFP are encouraging for photon down‐conversion. Therefore, we followed the procedure previously described using hydroxypropyl cellulose (HPC)^[^ ^14^ ^]^ to fabricate self‐standing dome‐shaped coatings (9 mm diameter × 4 mm height × 2 mm thickness) with the equivalent amounts of both green/red species of the QuetzalFP and EosFP (1 mg; see Supporting Information for details). In general, these coatings were colorful to the naked eye (**Figure** **4**). The emission and excitation band shapes are broadened (Figure 4A,B), while the thermal behavior shows a similar steady decrease of the emission intensity with the increase in temperature for both proteins (Figure 4C,F; Table 1). This behavior has been typically attributed to the formation of aggregated‐like protein structures upon film drying. This is further confirmed by the broad excitation spectra (Figure 4A,B).^[^ ^14^, ^16^, ^19^, ^20^, ^22^, ^23^, ^24^, ^25^, ^26^, ^27^ ^]^ This could result in a partial distortion of the protein structure within the matrix that might affect the overall H‐bonding network and the chromophore structure in the protein cavity. In general, a slight decrease in *ϕ* and an increase in *τ* compared to those in solution is noted (Table 1).^[^ ^14^, ^16^, ^19^, ^20^, ^22^, ^23^, ^24^, ^25^, ^26^, ^27^ ^]^ In particular, the coatings with the green QuetzalFP and EosFP species showed high *ϕ* (Table 1), while the vibrational progression of the emission band shape nicely resembles those in solution despite the broadening of the emission spectra. However, the red coatings of QuetzalFP showed a significant change of the emission vibrational structure compared to that in solution. This suggests a chromophore conformational change upon dehydration and/or a large degree of protein agglomeration involving both, matured and non‐matured proteins (Figure 4B). However, further confirmation should be provided by an ultra‐fast and temperature‐dependent spectroscopic study of the excited state dynamics, supported by atomistic QM/MM/MD simulations in a polymer cage. Nevertheless, the spectroscopic and thermal features of the green‐ and red‐emitting coatings of QuetzalFP and EosFP are already promising for their application to photon down‐conversion in Bio‐HLEDs.

**Figure 5:**
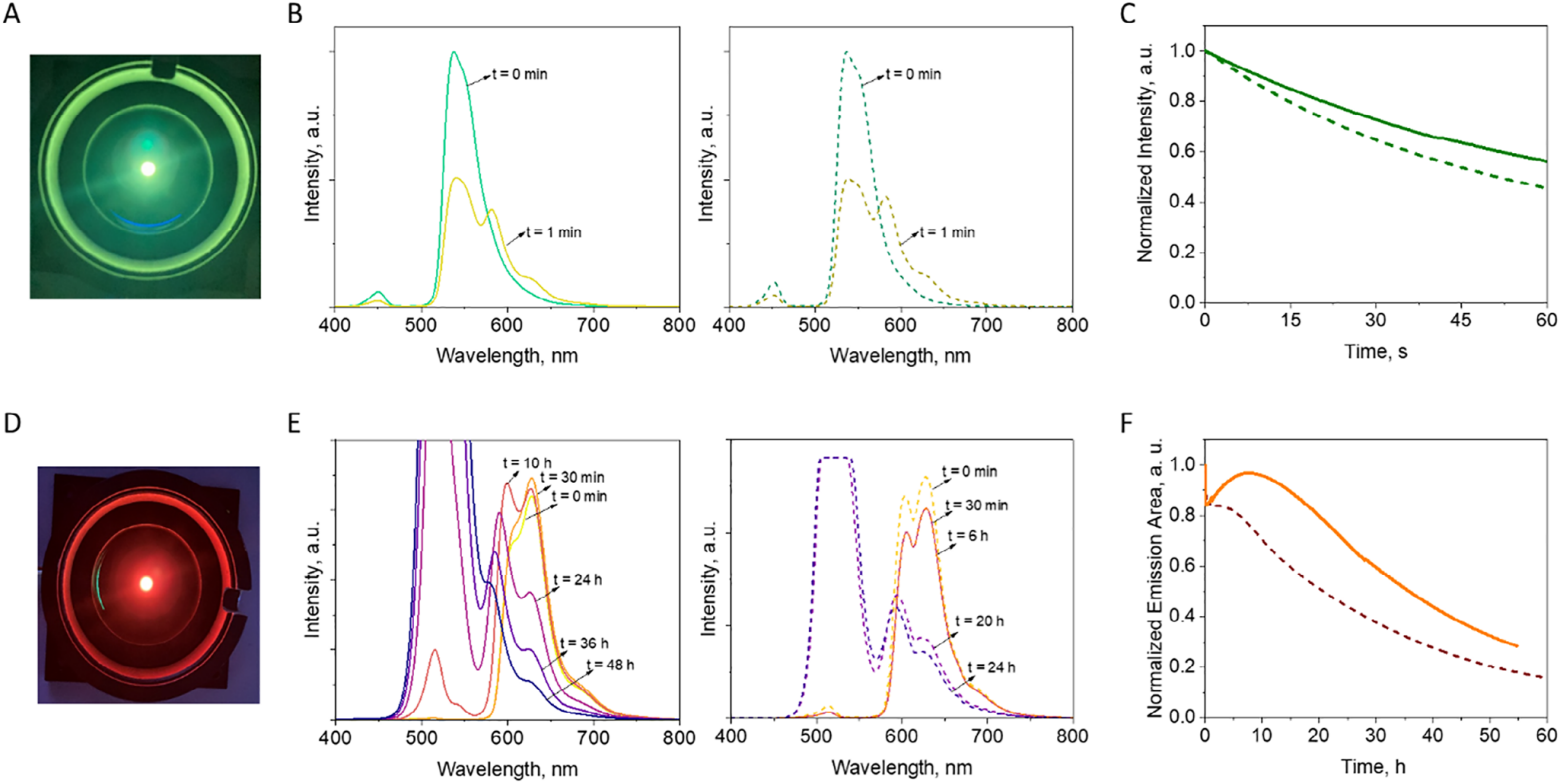
Characterization of QuetzalFP and EosFP Bio‐HLEDs. Pictures of green A) and red D) QuetzalFP devices under operation. Emission spectra of green B) and red E) emitting devices prepared with QuetzalFP (left) and EosFP (right) devices over time (see legend) at applied current of 200 mA. Device stability of green C) and red F) emitting devices of QuetzalFP (solid line) and EosFP (dashed line) species at an applied current of 200 mA. In the case of green emitting devices, the stability is related to the changes in the emission intensity band with respect to the initial value over time. In the red‐emitting devices, the stability is related to the changes of the emission area (570–750 nm) with respect to the initial value over time.

These devices were fabricated using commercial blue‐ (450 nm/1 W; Winger Electronics) and green‐ (520 nm/1 W; Winger Electronics) emitting LEDs that were directly covered with the above green‐ and red‐emitting protein‐HPC coatings, respectively (**Figure** **5**A and Supporting Information). The devices were driven at a constant current of 200 mA (240 mW/cm^2^ and 85 mW/cm^2^ for blue‐ and green‐emitting LEDs, respectively) to monitor the device stability with respect to the changes in the emission band (intensity and/or area) of each protein. Concerning the green‐emitting devices, both QuetzalFP and EosFP coatings led to a good conversion of the LED band with an emission band centered at 516 nm (Figure 5B) that corresponds to *x*/*y* CIE color coordinates of 0.36/0.57 (EosFP) and 0.35/0.56 (QuetzalFP). Unfortunately, the device stability and efficiency could not be properly studied, since the green emission intensity exponentially decays within the first minute as the emission features in the low‐energy region evolve due to the quick photoconversion process (Figure 5B,C). In addition, the device temperature rises up to 40 °C, suggesting the presence of thermally induced emission quenching (Figure S21, Supporting Information). Photoconversion is always present if other UV‐blue emitting LEDs are used (390, 420 and 470 nm) due to their intrinsic broad electroluminescence spectra. This prompted us to focus our efforts on the red QuetzalFP and EosFP based Bio‐HLEDs. Both red‐emitting devices showed a conversion of the green LED emission band associated to a low‐energy emission band covering the spectral range from 575 to 750 nm (Figure 5E) that corresponds to *x*/*y* CIE color coordinates of 0.64/0.36 (EosFP) and 0.67/0.33 (QuetzalFP). The device stability was carried out at 200 mA, monitoring the area changes of the down‐converting emission band as the relative intensity of the vibronic structure of the emission spectra changes over time (Figure 5C,F). This decay consists of three regimes, namely an initial exponential loss that is typically related to the increase in the temperature of the coating (Figure S21, Supporting Information) followed by a plateau/recovery stage, in which the emission band (shape and intensity) changes, and a final decay dominated by the photo‐induced degradation of the FPs related to ionic‐to‐neutral chromophore conversion, cis‐trans isomerization and/or oxidation.^[^ ^14^, ^16^, ^19^, ^20^, ^22^, ^23^, ^24^, ^25^, ^26^, ^27^ ^]^ Both red EosFP and QuetzalFP devices featured a similar first regime with an intensity loss of ≈20% associated to a temperature rise reaching up to ≈35 °C over the first minutes (Figure S21, Supporting Information). This was followed by a plateau stage for EosFP devices (Figure 5C,F), in which the emission spectra show a progressive change of the vibrational peak intensity ratios, suggesting a first photobleaching of the aggregated species over the non‐aggregated‐like ones. Once this process is over, a quick decay of the emission of the remaining species leads to a device stability of 18.5 h (Figure 5F). Here, the emission band shape does not change, suggesting a classical photo‐induced degradation of the protein chromophore. In stark contrast, red QuetzalFP devices exhibited a remarkable aggregation behavior as noted by the initial vibronic structure of the emission band and also a remarkable stability over ≈15 h (Figure 5F) associated to the above mentioned changes of the vibrational peak intensity ratios related to their progressive photobleaching. This is followed by a third decrease related to the degradation of the non‐aggregated species, without further changes of the shape of the emission spectra. Overall, this results in a device stability of 38 h (ca. 2‐fold enhancement, Figure 5F). This is in line with the features of the ancestral versus extant red variants with respect to a larger dimeric stabilization energy, a smaller chromophore binding pocket constraining geometrical distortions of the chromophore, and a simpler H‐bonding network around the chromophore (vide supra).

**Figure 6:**
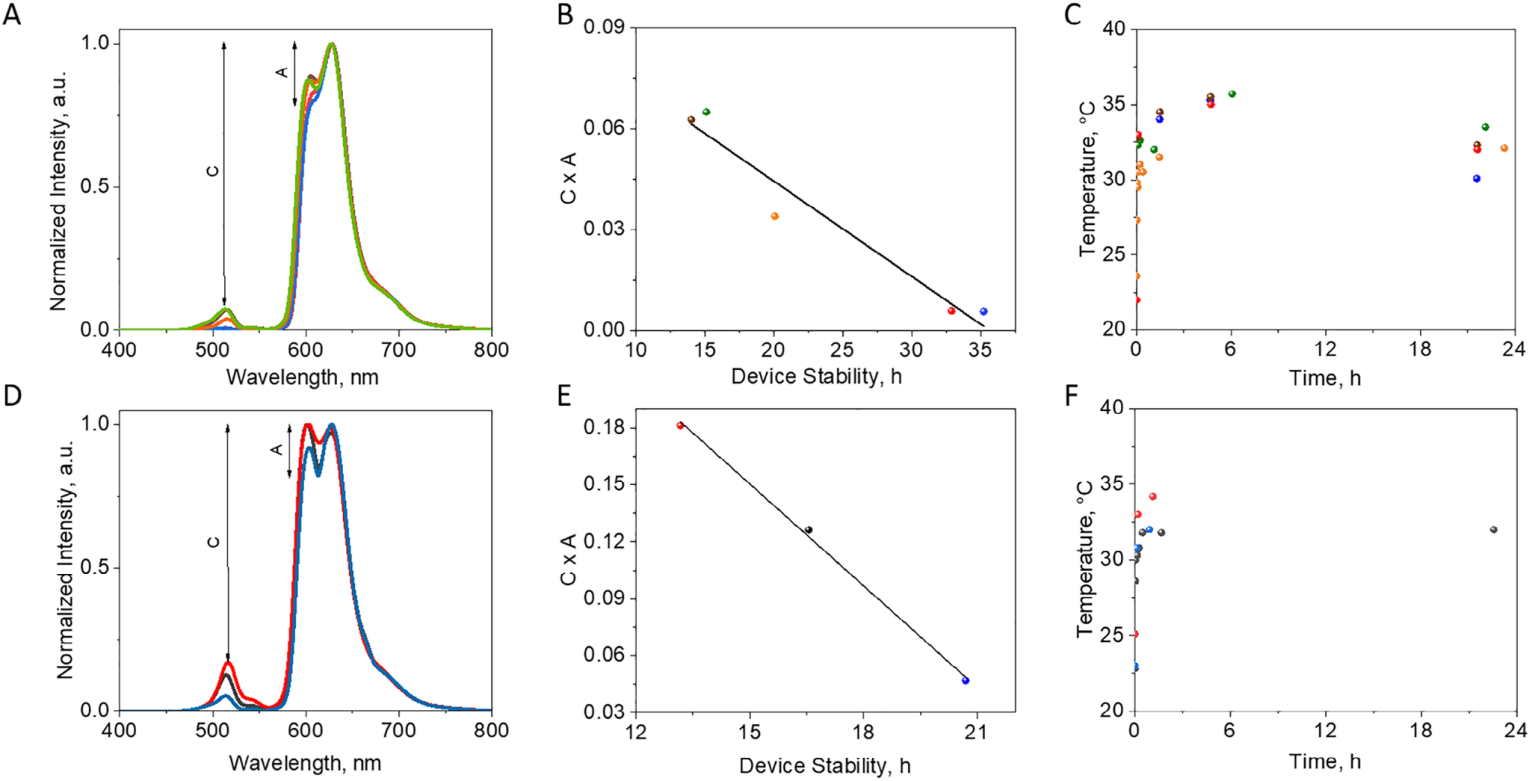
Characterization of QuetzalFP and EosFP Bio‐HLEDs. Fresh emission spectra of replicates of red emitting devices prepared with QuetzalFP A) and EosFP D) at an applied current of 200 mA, highlighting the parameters A (intensity ratio between first and second vibronic peaks of the protein emission band) and C (intensity ratio between the LED emission intensity and the highest intensity of the protein emission band). The empirical relation between the C × A and the device stability of QuetzalFP B) and EosFP E) at an applied current of 200 mA. The working temperature of the devices with QuetzalFP C) and EosFP F) at an applied current of 200 mA over time. The same color code is used for all the graphs for each respective device.

Since the aggregation phenomena in polymer coatings could be dependent of the amount of protein (non‐matured and matured), the blend of the protein‐polymer mixture and the coating drying process, we have prepared several replicates of the above red‐emitting devices for both proteins. As shown in **Figure** **6**A,D, we noted interesting changes in the first emission spectra with respect to the relative intensity of the vibronic peaks (A) and the conversion of the LED emission intensity band (C). Here, we noted two empirical relationships. On one hand, a higher aggregation tendency (high A) results in a better conversion (high C). On the other hand, the combined effect of both parameters (C × A) seems to be linearly correlated with the overall device stability in both proteins (Figure 6B,E), while the device working temperature is not altered by the aggregation behavior (Figure 6C,F). Thus, we can conclude that the higher stability of QuetzalFP might be putatively attributed to its higher tendency to aggregate in polymer coatings for a certain amount of protein.

## 3 Conclusion

This work puts forward a new methodology to discover and design ancestral‐like FPs for photon manipulation in lighting. This has been achieved by capitalizing on ASR, a quickly evolving method^[^ ^50^ ^]^ due to its dependence on different parameters, like the evolutionary models,^[^ ^45^ ^]^ algorithms,^[^ ^44^ ^]^ sequence alignments and/or phylogenetic tree topologies of the data set.^[^ ^44^, ^45^ ^]^ As our interests are technology oriented and many ancestral‐like proteins have already displayed superior properties,^[^ ^44^, ^49^ ^]^ this encouraged us to explore the discovery of FPs for optoelectronics regardless of the historical accuracy. Indeed, ASR appears robust enough to overcome epistasis problems^[^ ^69^ ^]^ that arise in regular protein engineering approaches involving mutant libraries, rational design or consensus design.^[^ ^49^, ^70^, ^71^ ^]^ In addition, this allows further optimization of the protein reconstruction taken into account alternative databases and algorithms.

In this line of rationale, QuetzalFP, the common ancestral‐like FP of the 221 members sharing interesting figures for lighting applications, has shown i) good bacteria production, ii) green‐ and red‐emitting forms showing remarkably high *ϕ* in solution (90% and 79% for green‐ and red‐emitting species, respectively), iii) excellent resilience in polymer coatings, fairly keeping the photoluminescence and thermal behaviors noted in solution and iv) red‐emitting Bio‐HLEDs with ≈2‐fold enhanced device stability compared to a reference protein, EosFP, possibly because QuetzalFP has a higher tendency to agglomerate due to its stronger dimeric structure, as suggested by joint experimental and QM/MM/MD simulations.

Overall, we envision that ASR‐based engineering is applicable to reshape protein‐based materials and technologies, since our results on ancestral FPs open a fresh way toward advancing sustainable FP‐optoelectronics for lighting. Indeed, not only the last common ancestral‐like protein QuetzalFP has disclosed a new starting point in the design of FPs for Bio‐HLEDs, but also the ASR approach has provided us with up to 219 possible ancestral‐like FPs (evolutionary nodes) of high interest for optoelectronics, photonics, and medical (phototherapy, bioimaging, sensing) applications. They represent the focus of our current efforts to identify the glimpses of the past toward the bright future of Bio‐HLEDs.

## Conflict of Interest

The authors declare no conflict of interest.

## Supporting information



Supporting Information

## Data Availability

The data that support the findings of this study are available from the corresponding author upon reasonable request.
