# Supplementary material for: Ancestral Protein‐Based Lighting
Source: Adv Mater. 2025 Jun 25;37(35):2420303. doi: 10.1002/adma.202420303 (PMC12412002; doi:10.1002/adma.202420303)
Supplement: Supplementary file 1 — Supporting Information [file ADMA-37-2420303-s001.docx]

Supporting Information

**Ancestral Protein-based Lighting**

*Stephanie Willeit,^†a^ Alexander Mauz,^†a^ David Gutiérrez-Armayor, ^a^ Joseph Arbash,**^a^ Jesús Agustín Banda-Vázquez,^a^* Sergio Martí,^b^* Pedro B. Coto,^c^* and Rubén D. Costa^a^**

^a^Technical University of Munich, Campus Straubing for Biotechnology and Sustainability, Chair of Biogenic Functional Materials, Schulgasse, 22, Straubing, 94315, Germany.

E-mail: ja.banda@tum.de, ruben.costa@tum.de

^b^Institute of Advanced Materials (INAM), Universitat Jaume I, Av. Vicent Sos Baynat, s/n, Castellón de la Plana 12071, Spain

E-mail: smarti@uji.es

^c^Nanomaterials and Nanotechnology Research Center (CINN), Spanish National Research Council (CSIC) and Donostia International Physics Center (DIPC), Avenida de la Vega, 4-6
El Entrego 33940, Spain

E-mail: pedro.brana@csic.es

**Sequence search and ASR:** The sequences of the following 22 known FPs: 22G,^[1]^ aceGFP,^[2]^ amFP486,^[3]^ anm2CP,^[4]^ avGFP,^[5]^ AvicFP1,^[6]^ cFP484,^[3]^ CpYGFP,^[7]^ dendFP,^[8]^ dfGFP,^[9]^ DrCBD,^[10]^ DsRED,^[3]^ EosFP,^[11]^ eqFP578,^[12]^ eqFP611,^[13]^ HcRed,^[14]^ KikG,^[15]^ KO,^[16]^ LanYFP,^[17]^ Montispora sp. #20,^[18]^ mRed7^[19]^ and zFP538^[3]^ were globally aligned with MAFFT online.^[20]^ A hidden Markov model (HMM) out of this MSA was built with the tool *hmmbuild* from the HMMER v3.3.2 suite,^[21]^ as a query to look into the UniRef100 database (August 3^rd^ 2022 version) for more homolog sequences, as described in https://www.uniprot.org/uniref/, the UniRef100 database contains all records in the Universal Protein Resource Knowledge database (UniProtKB), which is the central access point for extensively curated protein information, including function, classification and cross-references, which are organized in clusters to speed up sequence similarity searches, as part of a collaboration between the European Bioinformatics Institute (EBI), the Swiss Institute of Bioinformatics (SIB), and the Protein Information Resource (PIR). From this moment on, this approach followed the same steps as the one used in previous studies.^[22, 23]^ Briefly, 1008 hits were filtered with CD-HIT^[24]^ into a final set of 221 sequences, with the criteria that they were at least 200 residues long and none of them shared beyond 90% identity to any other. The final set was locally aligned to the previously calculated HMM with the tool *hmmalign* from HMMER v3.3.2. This local MSA was used for ASR on ANCESCON^[25]^ within the MPI Bioinformatics Toolkit,^[26, 27]^ and a phylogenetic tree was inferred, where the ancestral sequence for each of the 219 internal nodes was calculated. QuetzalFP was exceptionally interesting because it was the apparent last common ancestor on the tree that was automatically generated with the ANCESCON tool we used, as the only acceptable input was the MSA with no further specifications. As ANCESCON renamed the sequences with numbers starting by 0, in order to have a visual clue of the colors in the phylogeny, the names were changed by matching with the Basic Local Alignment Search Tool BLAST (https://blast.ncbi.nlm.nih.gov) these sequences against the initial 22 sequences we used to build the HMM; where the best match was used as a name. For example, the fraction of the sequence UniRef100_A8CLU1 that satisfies the HMM corresponds to the 135^th^ sequence in the input MSA, this sequence was internally renamed as Sequence134 in ANCESCON, then we renamed it as 135_amFP486_c, since amFP486, which is a protein with reported cyan fluorescence (*λ*_Em_ 486 nm),^[3]^ was the top match to Sequence134. Interestingly, none of these final hits showed a best match to DrCBD, possibly because it is the only protein that is not a β-barrel FP in the initial MSA.

**Alternative ASR:** The 6 sequences belonging to synthetic FPs labeled as synthetic in the MSA were UniRef100_UPI0002B7BE02 (an ancestral GFP-like protein,^[28]^ it is leaf 10_dendFP_gr in our tree at **Figure S1A**), UniRef100_UPI00051FF421 (also known as the thermostable eCGP123,^[29]^ 15_dendFP_gr), UniRef100_UPI000225A889 (an mKate variant,^[30]^ 58_eqFP578_o), UniRef100_UPI001A9A2C15 (the fluorescent probe POLArISact,^[31]^ 145_avGFP_g), UniRef100_UPI0006AB76F2 (the tryptophan-based chromophore FP nowGFP,^[32]^ 146_avGFP_g) and UniRef100_UPI000745F255 (another GFP mutant,^[33]^ 150_avGFP_g). As explained in the main manuscript, the removal of these sequences from the original MSA gave place to exactly the same sequence at the last common ancestor of the new phylogeny through ANCESCON as QuetzalFP.

**Material for the ASR reconstruction**: the 22 seed sequences, along with their MSA, the subsequent HMM, the MSA for the found hits before and after filtering, the ANCESCON outputs, including the trees before and after renaming the leaves names as in **Figure 1** are available upon request.

**Chemicals**: Chemical reagents were obtained from Sigma-Aldrich. Unless specified otherwise. Phosphate buffered saline (PBS) was used for all Protein experiments. PBS contained: Na2HPO4 1.42 g/L, KH2PO2 0.27 g/L, KCl 0.2 g/L, NaCl 8 mg/L, MilliQ quality double deionized water at pH 7.5. The NaCl was manufactured by VWR chemicals.

**Genetic constructs:** Codon-optimized genetic constructs for QuetzalFP and EosFP were ordered from TWIST bioscience with the coding sequence (CDS) under the control of a T7 expression system in pET29b(+) plasmids encoding Kanamycin resistance.

**Protein production and purification:** An *Escherichia coli (E. coli)* BL21 DE3 strain chassis was employed and transformed with the corresponding plasmids for protein production. The *E. coli* cells were cultivated in Lysogeny broth (LB) at 30 °C, with appropriate antibiotics until an OD_600_ of 0.4-0.6, and then induced with a final concentration of 1 mM IPTG (Isopropyl β-D-1-thiogalactopyranoside) after induction cells were moved to 16 °C, cultivated overnight and harvested the next day. The resulting cell pellet was harvested by centrifugation (4000 x g, 4 °C, 30 min) and then washed with PBS, recentrifuged (4000 x g, 4 °C, 30 min) and frozen at -20 °C. For purification, the frozen cell pellets containing QuetzalFP were thawed and resuspended in 50 mM Tris, 300 mM NaCl, 1 % Triton, 5 % Glycerol and 5 mM Ethylenediaminetetraacetic acid (EDTA) at pH 7.2. To this suspension lysozyme (Lysozyme HCl GERBU Biotechnik GmbH) was added. The resulting suspension was digested at room temperature for 90 min in darkness, under constant agitation, by inversion in a tube rotator. For EosFP, the frozen cells were simply resuspended in PBS. After sonication, the cell debris was removed by centrifugation (38758 x g at 4 °C for 1 h in a SORVAL Lynx 4000 (Thermo Scientific)). The resulting solution was purified by nickel affinity (HisTrap^TM^ in an Äkta pure (cytiva)), followed by desalting into PBS by size exclusion chromatography in a (HiLoadTM 26/600 SuperdexTM 75 pg, cytiva). After purification, the resulting protein solution in PBS was concentrated, frozen in liquid N_2_ and stored at -80 °C until use.

**Size exclusion chromatography:** The oligomerization behavior of the tested proteins was determined by size exclusion chromatography (SEC). This was done by loading specified protein concentrations onto the analytical grade SEC column (SuperdexTM 75 Increase 10/300 GL, by cytiva), on the FPLC (Äkta pure cytiva).

**Protein photo conversion:** QuetzalFP was photo converted for use in experiments by irradiating an aliquot of QuetzalFP in green state in a 1.5 mL micro volume tube with a 385 nm conal laser (Olis) until full conversion could be observed in the absorbance spectrum. The same was done for EosFP (**Figure S3**).

**Protein-polymer coating preparation**: HPC was dissolved to a concentration of 230 mg/mL in MilliQ water. For each coating, an amount of 1 mg of the respective FP was used. The FP and the HPC solutions were mixed at 100 rpm for 10 min, dome-shaped (9 mm diameter × 4 mm height × 2 mm thickness) and subsequently dried with vacuum. Coatings were stored at 4°C before usage.

**Photophysical characterization:** Photophysical studies, including absorption spectroscopy were carried out at ambient conditions. Absorption spectra of thawed protein solutions were recorded with a UV–vis-2600i spectrophotometer (Shimadzu). Photophysical studies were performed in an FS5 Spectrofluorometer (Edinburgh Instruments) with the respective sample holders (SC-5 for liquid, SC-10 for solids). Picosecond pulsed diode lasers EPL-375 and EPL 450 (Edinburgh Instruments) were used to determine the excited state lifetimes (τ). The photoluminescence quantum yield (φ) was determined by a Quantaurus-QY Absolute PL Quantum yield spectrometer C11347 (Hamamatsu). The extinction coefficient (ε) was determined as previously published^[34]^ using a UV–vis-2600i spectrophotometer (Shimadzu) (**Figure S4**).

**Thermocycler-enabled modulated scanning fluorimetry:** Protein melting studies were performed on a C1000TM thermal cycler base (Bio-Rad) with a CFX96TM Real-time PCR detection system (Bio-Rad). A program was set to start at 25°C and continue up to 99 °C. The temperature was raised by 1 °C per step (heating speed: 5 °C/s), to measure the progressive loss of fluorescence intensity. At the end of each heating period (held for 1 min), the fluorescence was measured and cooled back to 25 °C for another fluorescence measurement after a recovery period of 5 min. The melting curve was determined by the fluorescence at the hot temperature, while the refolding curve was determined using the fluorescence intensity after the recovery period. T_m_ refers to the melting temperature of the protein and it is defined as the temperature value at which the intensity of the emission is reduced to half of the initial value upon increasing temperature stress. T_nr_ refers to the non-reversible temperature and it is defined as the temperature at which the protein's structure changes irreversibly, affecting to its original emission intensity upon cooling. The value reported is the temperature value at which the intensity of the emission is reduced to half of the initial value

**CPL measurements:** As done in a previous work,^[35]^ the data was recorded using a Olis CPL Solo (Circulary polarized luminescence) spectrometer and the software OlisGlobalWorks. The raw data in arbitrary units were processed by an in-house Python script that normalized, averaged and processed the data with a Savitzky-Golay filtering of the repeated measurements. The final CPL curves were fit by the least-squares method using the LMFIT and SciPy Python toolboxes to the best double gaussian.

**Device fabrication and characterization:** The devices were fabricated using unmodified commercial 450 nm and 520 nm LEDs (WINGER® WEPYE1-S1 Power LED Star) for green and red Bio-HLEDs, respectively. The power of the LEDs were measured with a PM100D device with a S121C detector from THORLABS. To carry out the photostability studies, the protein-HPC coatings were directly placed onto the above commercial LEDs without any further modification, referred as on-chip in previous works in BioHLEDs.^[36-45]^ They were driven at 200 mA for long-term stability at ambient conditions. The emission spectra were recorded through an Avantes Spectrometer (300 VA grating, 200 µm slit, CCD detector) coupled with an AvaSphere-30-IRRAD, monitoring the temperature using a thermographic camera FLIR ETS320. The employed power source was a Keithley 2231-A-30-3.

**Figure S1.** Phylogenetic tree of the 221 hits and structural modelling. (A) Whole phylogenetic tree of the hits to the query MSA in this study (see text for details), where the last common ancestral-like node (blue circle) is shown in the middle. (B) Alternative tree where the sequences annotated as “synthetic” were removed. Where the last common ancestral-like node (blue circle in the middle) has exactly the same sequence as in A).


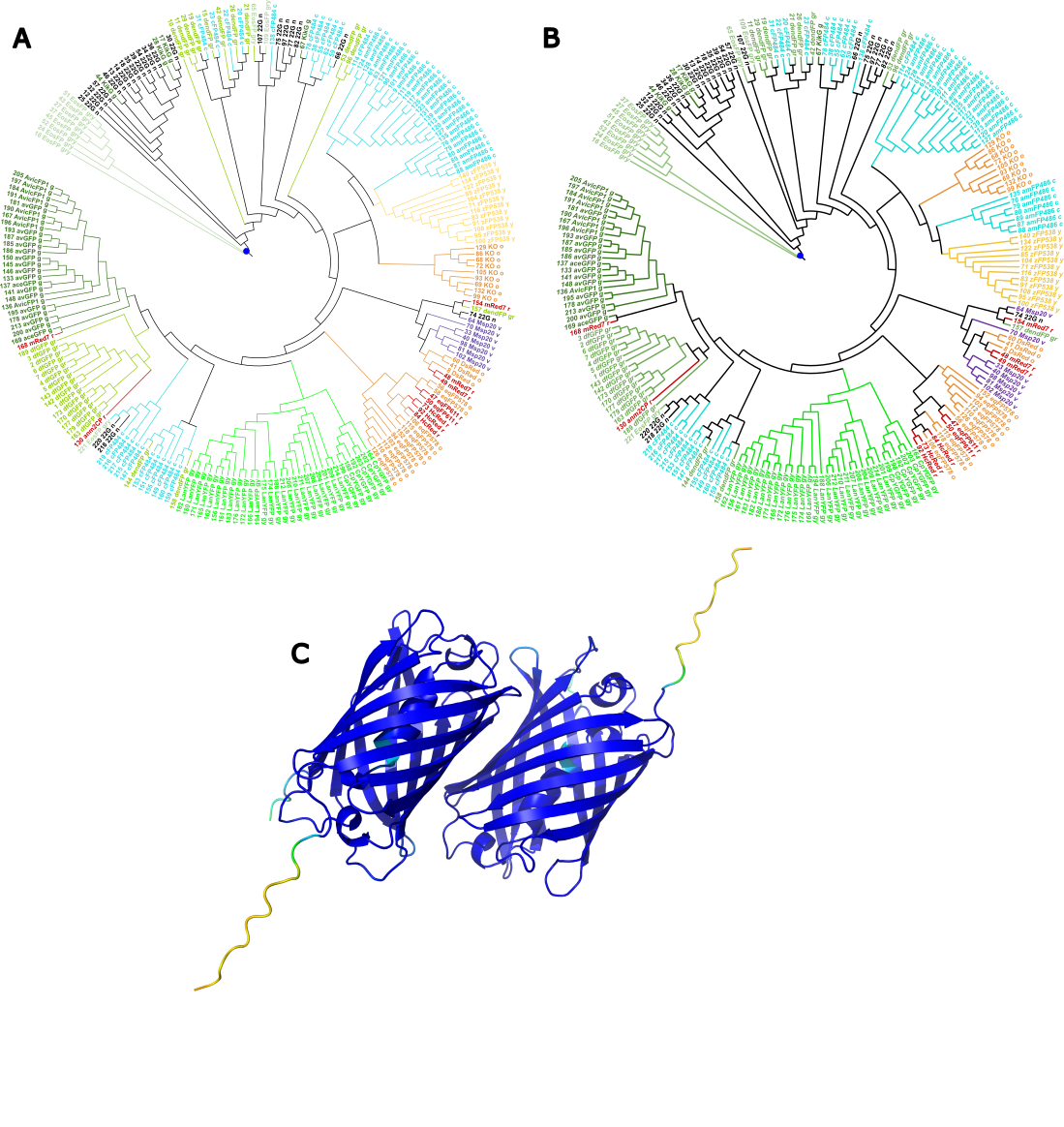


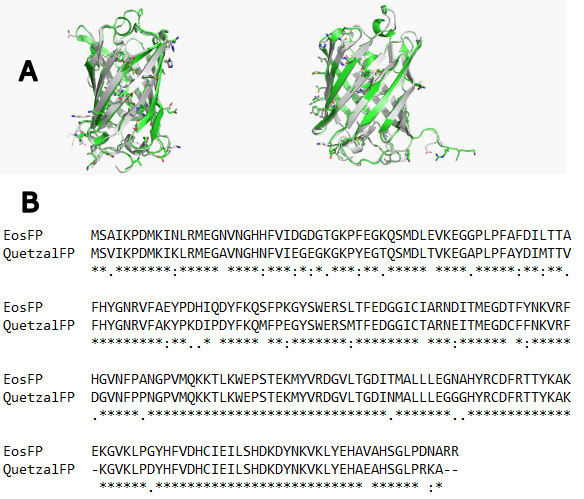


**Figure S2.** **Sequence comparison between QuetzalFP and EosFP.** MAFFT^[20]^ pairwise sequence alignment of both proteins, with identical residues marked with (*), conserved substitution with (:), semiconserved substitutions with (.) and no matching comparisons with (-).


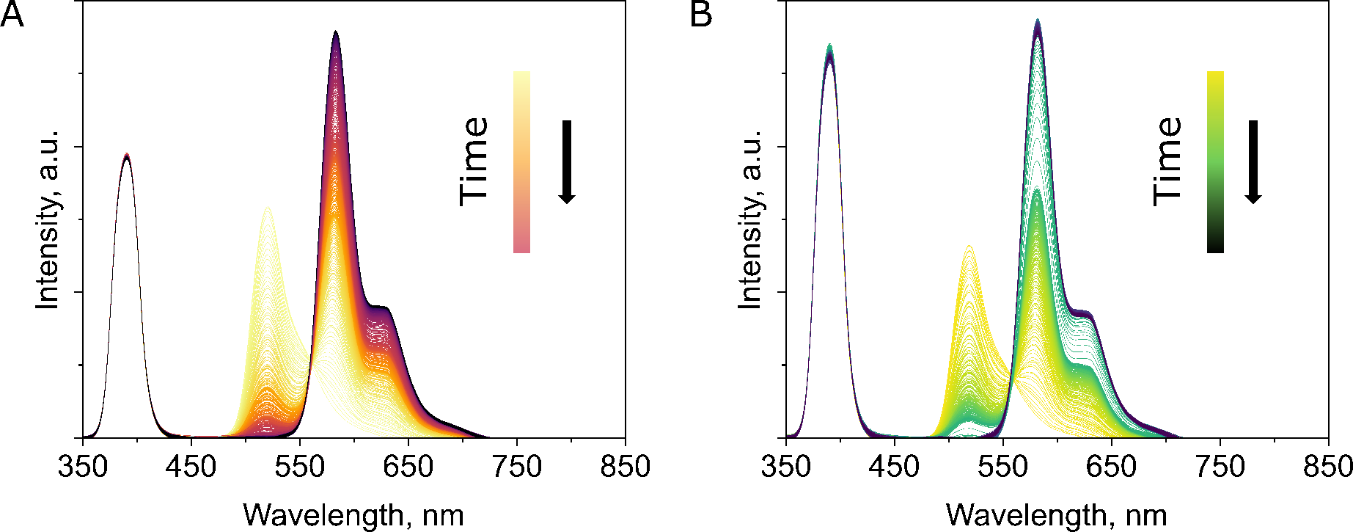


**Figure S3**. Photoconversion of QuetzalFP (A) and EosFP (B) at constant 385 nm excitation.


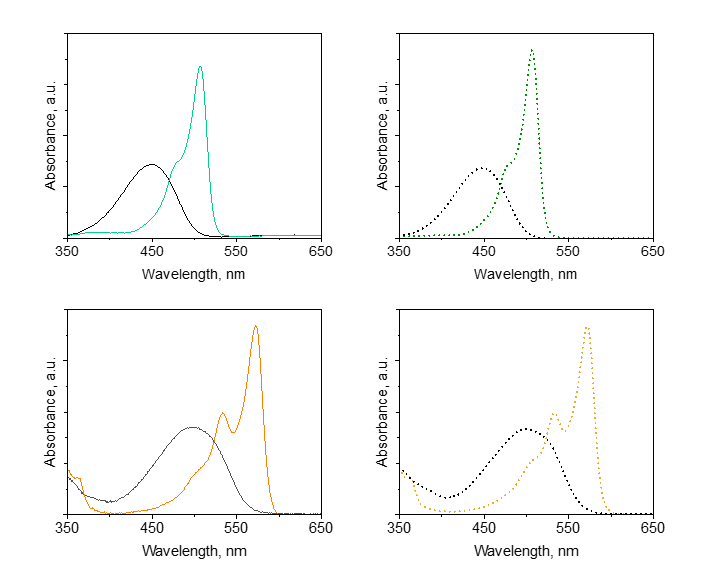


**Figure S4**. Extinction coefficient determination of QuetzalFP (left) and EosFP (right) in the green (top) and the red (bottom) species. The absorption spectra in NaOH is provided in black.


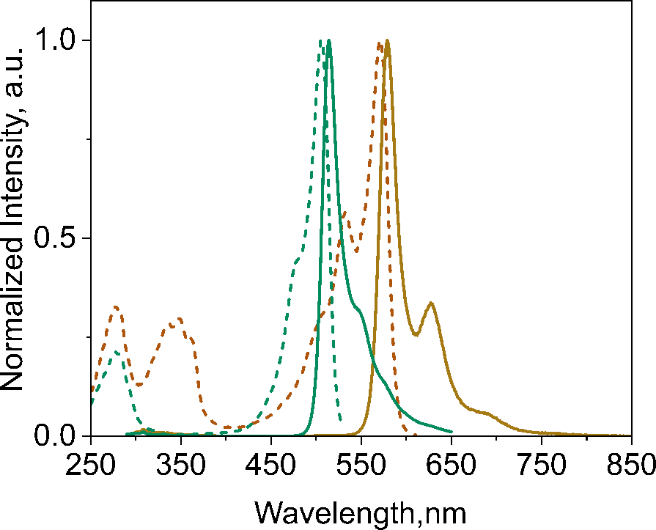


**Figure S5**. Excitation (dashed line) and emission (solid line) spectra of green (dark green) and red (dark orange) species of EosFP.


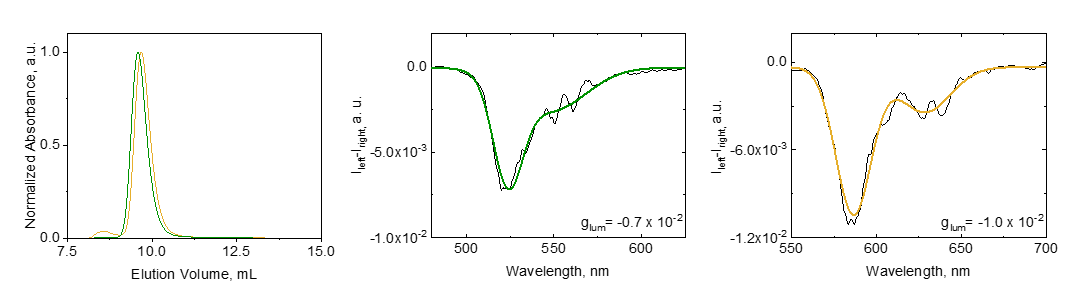


**Figure S6**. Analytical SEC (left) and the CPL (center/right) spectra in solution of the green and red species of EosFP.


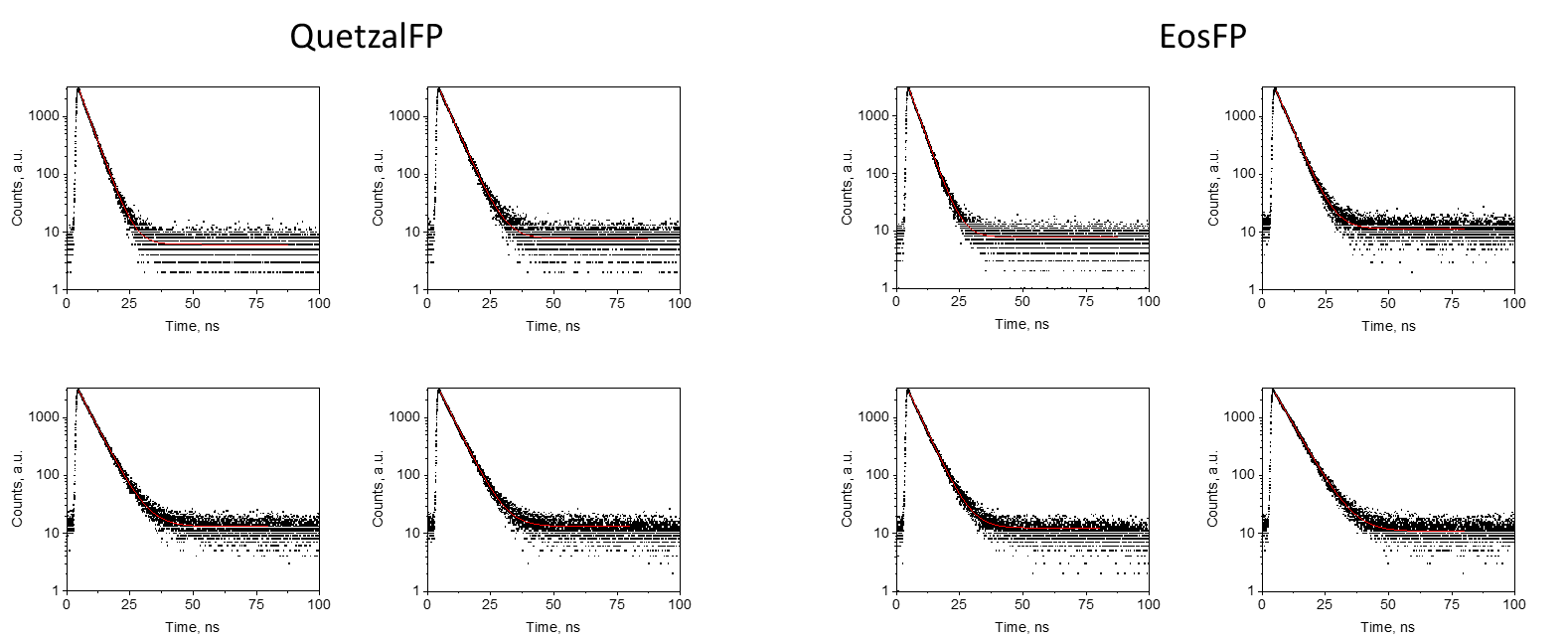


**Figure S7**. Excited state lifetime decay (black) and the fittings (red) of the respective proteins in solution (top) and HPC coatings (bottom) of green (left) and red (right) forms of QuetzalFP and EosFP.

**
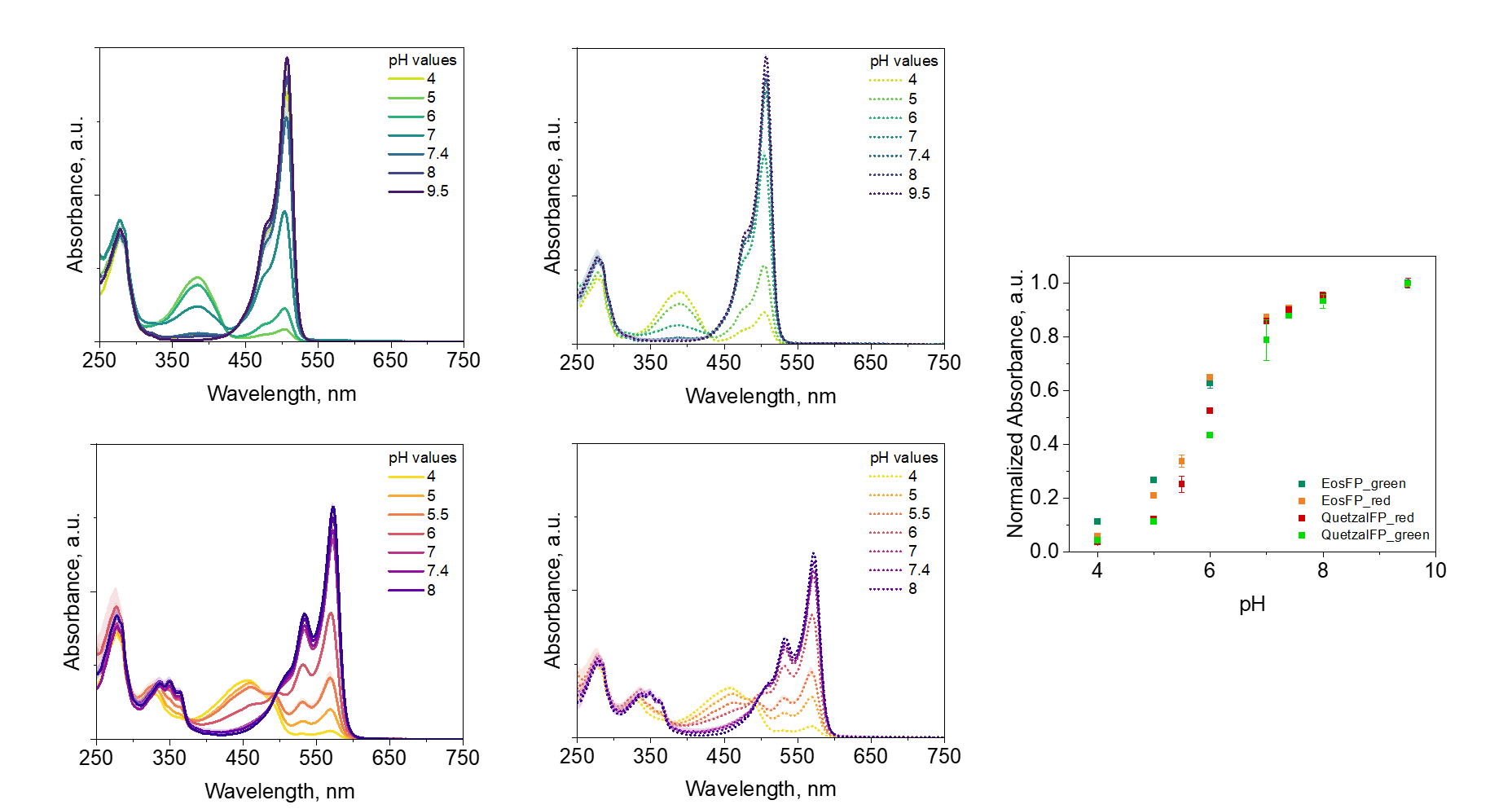
**

**Figure S8**. Absorption spectra changes of QuetzalFP (left) and EosFP (right) for the green (top) and red (bottom) species in aqueous solution upon changing the pH (see legend). The graphs on the right side show the changes in the maximum absorption wavelength upon changing the pH. The values are calculated as the mean of 3 samples.

**
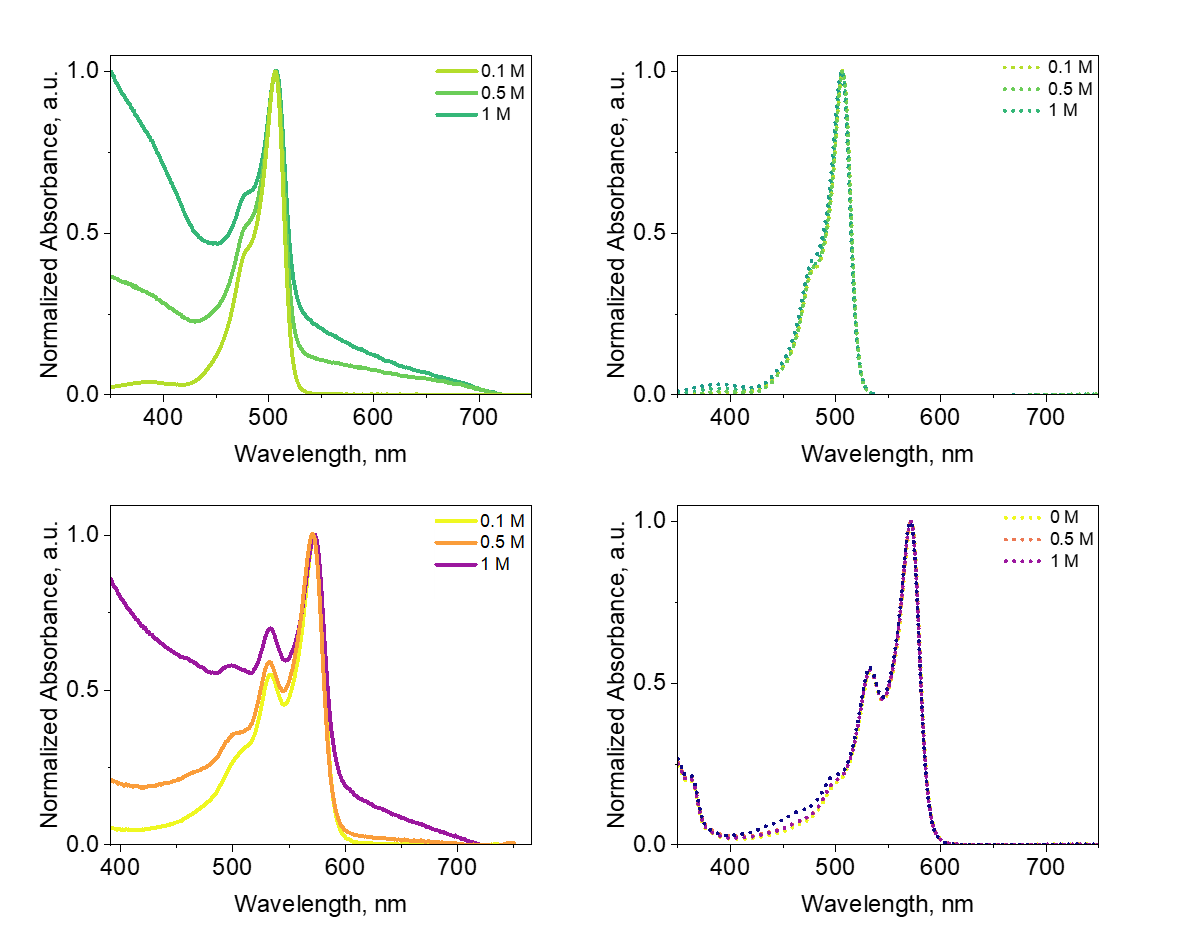
**

**Figure S9**. Absorption spectra changes of QuetzalFP (left) and EosFP (right) for the green (top) and red (bottom) species upon increasing the amount of NaCl in buffer solution (see legend).

**Discussion S1: Molecular dynamics simulations.** The setup of both fluorescent proteins was carried out using the AMBER ff03 forcefield,^[46]^ starting from the corresponding X-ray structures (see main text). For EosFP we used as starting structures those found in the PDB codes 1ZUX for the green active version, and 2BTJ for the red one (both structures differ in the first two resolved amino acids: MET-SER in the green and GLY-GLY in the red). On the other hand, we used an internally resolved X-ray structure for a QuetzalFP variant (still unpublished). In both cases, we considered the dimeric state of the proteins to setup the computational models. The green active HYG chromophore naturally present in the EosFP was parametrized using *Antechamber*^[47]^ utility available in AmberTools (v20),^[48]^ and the parameters were taken from the generalized AMBER forcefield (GAFF).^[49]^ This structure was superimposed in the active site of QuetzalFP, which was initially resolved with the TYG chromophore variant. The red active chromophore present in the EosFP X-ray structure was modelled as proposed in the literature^[50]^, and again manually superimposed in the QuetzalFP structure. The pKa of the tritatable aminoacids was determined using the heuristic program PROPKA-3.5.1 ^[51, 52]^ at pH 7. The results obtained were consistent with the standard protonation state for all the titratable amino acids except for three doubly protonated histidine residues in the EosFP (His-22, 194 and 217 in both subunits), and two protonated histidines (His-196 and 219 in both subunits) in the QuetzalFP. To obtain a neutral system, the charge of EosFP was neutralized by the addition of six Na^+^ cations, meanwhile QuetzalFP was neutralized by the addition of two Cl^-^ anions. No ionic strength was added. All models were solvated with TIP3P water molecules^[53]^ 15 Å long on each axis around the proteins (tLeap command: “solvatebox complex TIP3PBOX 15.0 0.8”) producing orthorhombic boxes with dimensions of approximately 104x86x76 Å^3^. Using these models as starting structures we initially carried out 1 ns of classical MD at 300 K and 1 atm in the NPT ensemble, with a switched cutoff of 12 to 14 Å for the long-range interactions together with the particle-mesh Ewald (PME) method, an integration time step of 1 fs and using the Langevin piston barostat and thermostat, as implemented in NAMD (v3.0b6).^[54]^ Once the size of the systems was equilibrated, we ran 1 μs at 300 K in the NVT ensemble employing the same parameters but with an integration time step of 2 fs, and using the Langevin thermostat as implemented in OpenMM (v8.1.0).^[55]^ Finally, an additional QM/MM NVT MD run of 1.1 ns was performed for each model using the DFTB3 Hamiltonian^[56–58]^ along with the 3OB set of parameters^[59]^ as implemented in the *Sander* program. For convenience, only one of the chromophores present in each model was described at the QM level, while the rest of the system was described at the MM level. All the results presented below correspond to the analysis of the monomeric unit with the chromophore described at the QM level for these last MD runs, unless otherwise stated. Regarding the selection of parameters and simulation conditions used in this study (such as cut-offs, integration steps, order of application of the ensembles, etc.), they have been selected to fit the usual options in the research field (see for example publications by S. Martí et al.).

**Table S1**. Atom types, charges (in a.u.) and parameters for the green HYG chromophore, using the GAFF force field

C12-H26 C6-H27 C4-H28 C5-H29 C3-H31 C23-H32 C10-H33 C20-H35 C20-H36

N1-H39 CA1-H40 CA3-H41 CA3-H42

| **LABEL** | **TYPE** | **CHARGE** |  | **LABEL** | **TYPE** | **CHARGE** |  |  |
| --- | --- | --- | --- | --- | --- | --- | --- | --- |
| N1 | N | -1.01177 |  | C7 | CA | -0.022717 |  |  |
| H38 | H | 0.402922 |  | C5 | CA | 0.047666 |  |  |
| CA1 | CT | 0.736962 |  | H29 | HA | 0.108803 |  |  |
| H40 | H1 | -0.075621 |  | C4 | CA | -0.553727 |  |  |
| C20 | CT | -0.659204 |  | H28 | HA | 0.176957 |  |  |
| H35 | HC | 0.181951 |  | C11 | C | 0.865933 |  |  |
| H36 | HC | 0.210214 |  | C12 | CA | -0.506902 |  |  |
| C21 | CC | 0.268307 |  | H26 | HA | 0.166299 |  |  |
| N22 | NA | -0.500231 |  | C6 | CA | -0.087853 |  |  |
| H37 | H | 0.394077 |  | H27 | HA | 0.14138 |  |  |
| C10 | CR | 0.26993 |  | O13 | O | -0.718515 |  |  |
| H33 | H5 | 0.111249 |  | C2 | C | 0.50556 |  |  |
| N11 | NB | -0.572069 |  | O2 | O | -0.644568 |  |  |
| C23 | CV | 0.06795 |  | N3 | N* | -0.1662 |  |  |
| H32 | H4 | 0.118677 |  | CA3 | CT | -0.068269 |  |  |
| C1 | CK | 0.053213 |  | H41 | H1 | 0.033988 |  |  |
| N2 | NB | -0.375671 |  | H42 | H1 | 0.135403 |  |  |
| CA2 | CC | -0.024987 |  | C3 | C | 0.517142 |  |  |
| C8 | CD | -0.166206 |  | O3 | O | -0.517947 |  |  |
| H30 | HA | 0.157874 |  |  |  |  |  |  |
|  |  |  |  |  |  |  |  |  |
|  |  |  |  |  |  |  |  |  |
| **BOND** |  |  |  | **DIHE** |  |  |  |  |
| H-N | 403.2 | 1.013 |  | HC-CT-CT-N | 9 | 1.4 | 0 | 3 |
| CT-N | 328.7 | 1.462 |  | CC-CT-CT-N | 9 | 1.4 | 0 | 3 |
| CT-H1 | 330.6 | 1.097 |  | NB-CK-CT-N | 6 | 0 | 0 | 3 |
| CT-CT | 300.9 | 1.538 |  | N*-CK-CT-N | 6 | 0 | 0 | 3 |
| CK-CT | 334.8 | 1.502 |  | H1-CT-N-H | 6 | 0 | 0 | 2 |
| CT-HC | 330.6 | 1.097 |  | CT-CT-N-H | 6 | 0 | 0 | 2 |
| CC-CT | 334.8 | 1.502 |  | CK-CT-N-H | 6 | 0 | 0 | 2 |
| CC-NA | 425.8 | 1.38 |  | NA-CC-CT-CT | 6 | 0 | 0 | 3 |
| CC-CV | 419.8 | 1.428 |  | CV-CC-CT-CT | 6 | 0 | 0 | 3 |
| H-NA | 408.4 | 1.01 |  | CT-CK-NB-CC | 2 | 9.5 | 180 | 2 |
| CR-NA | 425.8 | 1.38 |  | CT-CK-N*-C | 2 | 9.5 | 180 | 2 |
| CR-H5 | 351.7 | 1.082 |  | CT-CK-N*-CT | 2 | 9.5 | 180 | 2 |
| CR-NB | 441.1 | 1.369 |  | H1-CT-CT-HC | 9 | 1.4 | 0 | 3 |
| CV-NB | 441.1 | 1.369 |  | CC-CT-CT-H1 | 9 | 1.4 | 0 | 3 |
| CV-H4 | 352 | 1.082 |  | NB-CK-CT-H1 | 6 | 0 | 0 | 3 |
| CK-NB | 441.1 | 1.369 |  | N*-CK-CT-H1 | 6 | 0 | 0 | 3 |
| CK-N* | 441.1 | 1.369 |  | NB-CK-CT-CT | 6 | 0 | 0 | 3 |
| CC-NB | 441.1 | 1.369 |  | N*-CK-CT-CT | 6 | 0 | 0 | 3 |
| CC-CD | 419.8 | 1.428 |  | CT-CC-NA-H | 4 | 6.8 | 180 | 2 |
| C-CC | 371 | 1.468 |  | CT-CC-NA-CR | 4 | 6.8 | 180 | 2 |
| CD-HA | 349.1 | 1.084 |  | CT-CC-CV-NB | 4 | 16 | 180 | 2 |
| CA-CD | 385.1 | 1.456 |  | CT-CC-CV-H4 | 4 | 16 | 180 | 2 |
| CA-CA | 461.1 | 1.398 |  | NA-CC-CT-HC | 6 | 0 | 0 | 3 |
| CA-HA | 345.8 | 1.086 |  | CV-CC-CT-HC | 6 | 0 | 0 | 3 |
| C-CA | 345.9 | 1.491 |  | H5-CR-NA-CC | 4 | 6.8 | 180 | 2 |
| C-O | 637.7 | 1.218 |  | NB-CR-NA-CC | 4 | 6.8 | 180 | 2 |
| C-N* | 416.9 | 1.387 |  | NA-CC-CV-NB | 4 | 16 | 180 | 2 |
| CT-N* | 334.7 | 1.456 |  | NA-CC-CV-H4 | 4 | 16 | 180 | 2 |
| C-CT | 313 | 1.524 |  | NA-CR-NB-CV | 2 | 9.5 | 180 | 2 |
|  |  |  |  | H5-CR-NA-H | 4 | 6.8 | 180 | 2 |
|  |  |  |  | NB-CR-NA-H | 4 | 6.8 | 180 | 2 |
| **ANGLE** |  |  |  | CC-CV-NB-CR | 2 | 9.5 | 180 | 2 |
| H1-CT-N | 49.8 | 108.88 |  | H4-CV-NB-CR | 2 | 9.5 | 180 | 2 |
| CT-CT-N | 65.9 | 111.61 |  | H5-CR-NB-CV | 2 | 9.5 | 180 | 2 |
| CK-CT-N | 66.7 | 111.76 |  | CV-CC-NA-H | 4 | 6.8 | 180 | 2 |
| CT-N-H | 45.8 | 117.68 |  | CV-CC-NA-CR | 4 | 6.8 | 180 | 2 |
| CT-CT-HC | 46.3 | 109.8 |  | CK-CT-CT-HC | 9 | 1.4 | 0 | 3 |
| CC-CT-CT | 63.5 | 111.93 |  | CC-CT-CT-CK | 9 | 1.4 | 0 | 3 |
| CT-CK-NB | 66 | 120.95 |  | CD-CC-NB-CK | 2 | 9.5 | 180 | 2 |
| CT-CK-N* | 66 | 120.95 |  | C-CC-NB-CK | 2 | 9.5 | 180 | 2 |
| CT-CT-H1 | 46.4 | 109.56 |  | H1-CT-N*-CK | 6 | 0 | 0 | 3 |
| CK-CT-H1 | 47.4 | 109.64 |  | C-CT-N*-CK | 6 | 0 | 0 | 3 |
| CK-CT-CT | 63.5 | 111.93 |  | NB-CK-N*-C | 2 | 9.5 | 180 | 2 |
| CT-CC-NA | 65.3 | 122.73 |  | NB-CK-N*-CT | 2 | 9.5 | 180 | 2 |
| CT-CC-CV | 64.6 | 115.97 |  | NB-CC-CD-HA | 4 | 16 | 180 | 2 |
| HC-CT-HC | 39.4 | 107.58 |  | NB-CC-CD-CA | 4 | 16 | 180 | 2 |
| CC-CT-HC | 47.2 | 110.49 |  | O-C-CC-NB | 4 | 11.5 | 180 | 2 |
| CC-NA-H | 46.8 | 125.5 |  | N*-C-CC-NB | 4 | 11.5 | 180 | 2 |
| CC-NA-CR | 68.5 | 109.9 |  | CA-CA-CD-CC | 4 | 2.8 | 180 | 2 |
| CC-CV-NB | 67.6 | 121.98 |  | CC-C-N*-CK | 2 | 8 | 180 | 2 |
| CC-CV-H4 | 45.9 | 127.96 |  | CC-C-N*-CT | 2 | 8 | 180 | 2 |
| CV-CC-NA | 68.6 | 117.77 |  | O-C-CC-CD | 4 | 11.5 | 180 | 2 |
| H5-CR-NA | 49.6 | 121.55 |  | N*-C-CC-CD | 4 | 11.5 | 180 | 2 |
| NA-CR-NB | 70.6 | 121.95 |  | CD-CA-CA-HA | 4 | 14.5 | 180 | 2 |
| CR-NA-H | 46.8 | 125.5 |  | CA-CA-CA-CD | 4 | 14.5 | 180 | 2 |
| CR-NB-CV | 71 | 103.76 |  | CA-CA-CD-HA | 4 | 2.8 | 180 | 2 |
| H5-CR-NB | 49.6 | 122.92 |  | CA-CA-CA-HA | 4 | 14.5 | 180 | 2 |
| H4-CV-NB | 50 | 121.14 |  | C-CA-CA-CA | 4 | 14.5 | 180 | 2 |
| CC-NB-CK | 71 | 103.76 |  | CA-CA-CA-CA | 4 | 14.5 | 180 | 2 |
| C-N*-CK | 66.7 | 120.49 |  | CA-C-CA-CA | 4 | 4 | 180 | 2 |
| CK-N*-CT | 67.9 | 109.51 |  | O-C-CA-CA | 4 | 4 | 180 | 2 |
| N*-CK-NB | 69.8 | 125.7 |  | HA-CA-CA-HA | 4 | 14.5 | 180 | 2 |
| CD-CC-NB | 67.6 | 121.98 |  | C-CA-CA-HA | 4 | 14.5 | 180 | 2 |
| C-CC-NB | 66.2 | 123.32 |  | CA-C-CA-HA | 4 | 4 | 180 | 2 |
| CC-CD-HA | 47.1 | 121.07 |  | O-C-CA-HA | 4 | 4 | 180 | 2 |
| CA-CD-CC | 67.2 | 111.04 |  | C-CC-CD-HA | 4 | 16 | 180 | 2 |
| CC-C-O | 69.1 | 123.93 |  | C-CC-CD-CA | 4 | 16 | 180 | 2 |
| CC-C-N* | 68.6 | 113.75 |  | H1-CT-N*-C | 6 | 0 | 0 | 3 |
| C-CC-CD | 63.6 | 122.69 |  | C-CT-N*-C | 6 | 0 | 0 | 3 |
| CA-CA-CD | 65 | 120.79 |  | O-C-N*-CK | 2 | 8 | 180 | 2 |
| CA-CD-HA | 45.8 | 124.04 |  | O-C-N*-CT | 2 | 8 | 180 | 2 |
| CA-CA-HA | 48.2 | 119.88 |  | N*-CK-NB-CC | 2 | 9.5 | 180 | 2 |
| CA-CA-CA | 66.6 | 120.02 |  | O-C-CT-N* | 6 | 0 | 180 | 2 |
| C-CA-CA | 64.3 | 120.33 |  | O-C-CT-H1 | 1 | 0.8 | 0 | -1 |
| CA-C-CA | 63 | 118.11 |  | O-C-CT-H1 | 1 | 0 | 0 | -2 |
| CA-C-O | 68.7 | 122.6 |  | O-C-CT-H1 | 1 | 0.08 | 180 | 3 |
| C-CA-HA | 46.4 | 115.9 |  |  |  |  |  |  |
| C-N*-CT | 67.9 | 109.51 |  |  |  |  |  |  |
| N*-C-O | 73.9 | 123.18 |  |  |  |  |  |  |
| H1-CT-N* | 50.1 | 108.57 |  |  |  |  |  |  |
| C-CT-N* | 68.1 | 106.51 |  |  | **NONBON** |  |  |  |
| CT-C-O | 67.4 | 123.2 |  |  |  | N | 1.824 | 0.17 |
| H1-CT-H1 | 39.2 | 108.46 |  |  |  | H | 0.6 | 0.0157 |
| C-CT-H1 | 47 | 108.22 |  |  |  | CT | 1.908 | 0.1094 |
|  |  |  |  |  |  | H1 | 1.387 | 0.0157 |
|  |  |  |  |  |  | HC | 1.487 | 0.0157 |
| **IMPROPER** |  |  |  |  |  | CC | 1.908 | 0.086 |
| CT-CV-CC-NA | 1.1 | 180 | 2 |  |  | NA | 1.824 | 0.17 |
| CC-CR-NA-H | 1.1 | 180 | 2 |  |  | CR | 1.908 | 0.086 |
| H5-NA-CR-NB | 1.1 | 180 | 2 |  |  | H5 | 1.359 | 0.015 |
| CC-H4-CV-NB | 1.1 | 180 | 2 |  |  | NB | 1.824 | 0.17 |
| CT-N*-CK-NB | 10.5 | 180 | 2 |  |  | CV | 1.908 | 0.086 |
| C-CD-CC-NB | 1.1 | 180 | 2 |  |  | H4 | 1.409 | 0.015 |
| CA-CC-CD-HA | 1.1 | 180 | 2 |  |  | CK | 1.908 | 0.086 |
| CA-CA-CA-CD | 1.1 | 180 | 2 |  |  | CD | 1.908 | 0.086 |
| CA-CA-CA-HA | 1.1 | 180 | 2 |  |  | HA | 1.459 | 0.015 |
| C-CA-CA-HA | 1.1 | 180 | 2 |  |  | CA | 1.908 | 0.086 |
| CA-CA-C-O | 10.5 | 180 | 2 |  |  | C | 1.908 | 0.086 |
| CC-N*-C-O | 10.5 | 180 | 2 |  |  | O | 1.6612 | 0.21 |
| C-CK-N*-CT | 1.1 | 180 | 2 |  |  | N* | 1.824 | 0.17 |

**Table S2**. Atom types, charges (in a.u.) and parameters for the red HYG chromophore (NFA, left panel) and RC7 (right panel), using the GAFF force field.

|  |  |
| --- | --- |
| CD1-H13 CE1-H14 CZ-H15 CD2-H16  CE2-H17 N-H19 CA-H21 CB-H22 CB-H23 | CE2-H25 CD2-H26 CD1-H27 CE1-H28  C3-H30 CA1-H31 CB1-H32 CD3-H33  CE3-H34 CA3-H35 CA3-H36 |

**NFA**:

| **LABEL** | **TYPE** | **CHARGE** |  | **LABEL** | **TYPE** | **CHARGE** |  |  |
| --- | --- | --- | --- | --- | --- | --- | --- | --- |
| N | N | -0.824354 |  | CG | ca | -0.075918 |  |  |
| H18 | H | 0.384154 |  | CD1 | ca | -0.10832 |  |  |
| CA | CT | 0.118882 |  | H13 | ha | 0.140206 |  |  |
| H21 | H1 | 0.081778 |  | CE1 | ca | -0.122327 |  |  |
| C | C | 0.647293 |  | H14 | ha | 0.139673 |  |  |
| O | O | -0.581848 |  | CZ | ca | -0.119526 |  |  |
| NXT | n | -0.626577 |  | H15 | ha | 0.139673 |  |  |
| H24 | hn | 0.332123 |  | CE2 | ca | -0.122327 |  |  |
| H25 | hn | 0.332123 |  | H17 | ha | 0.139673 |  |  |
| CB | CT | -0.027173 |  | CD2 | ca | -0.10832 |  |  |
| H22 | H1 | 0.060454 |  | H16 | ha | 0.140206 |  |  |
| H23 | H1 | 0.060454 |  |  |  |  |  |  |
|  |  |  |  |  |  |  |  |  |
|  |  |  |  |  |  |  |  |  |
| **BOND** |  |  |  | **DIHE** |  |  |  |  |
| H-N | 403.2 | 1.013 |  | O-C-CT-N | 6 | 0 | 180 | 2 |
| CT-N | 328.7 | 1.462 |  | n-C-CT-N | 1 | 1.7 | 180 | -1 |
| CT-H1 | 330.6 | 1.097 |  | n-C-CT-N | 1 | 2 | 180 | 2 |
| C-CT | 313 | 1.524 |  | H1-CT-CT-N | 9 | 1.4 | 0 | 3 |
| CT-CT | 300.9 | 1.538 |  | N-CT-CT-ca | 9 | 1.4 | 0 | 3 |
| C-O | 637.7 | 1.218 |  | H1-CT-N-H | 6 | 0 | 0 | 2 |
| C-n | 427.6 | 1.379 |  | C-CT-N-H | 6 | 0 | 0 | 2 |
| hn-n | 403.2 | 1.013 |  | CT-CT-N-H | 6 | 0 | 0 | 2 |
| CT-ca | 321 | 1.516 |  | CT-C-n-hn | 4 | 10 | 180 | 2 |
| ca-ca | 461.1 | 1.398 |  | CT-CT-ca-ca | 6 | 0 | 0 | 2 |
| ca-ha | 345.8 | 1.086 |  | O-C-CT-H1 | 1 | 0.8 | 0 | -1 |
|  |  |  |  | O-C-CT-H1 | 1 | 0 | 0 | -2 |
|  |  |  |  | O-C-CT-H1 | 1 | 0.08 | 180 | 3 |
| **ANGLE** |  |  |  | n-C-CT-H1 | 6 | 0 | 180 | 2 |
| H1-CT-N | 49.8 | 108.88 |  | H1-CT-CT-H1 | 9 | 1.4 | 0 | 3 |
| C-CT-N | 67 | 109.06 |  | H1-CT-CT-ca | 9 | 1.4 | 0 | 3 |
| CT-CT-N | 65.9 | 111.61 |  | C-CT-CT-H1 | 9 | 1.4 | 0 | 3 |
| CT-N-H | 45.8 | 117.68 |  | C-CT-CT-ca | 9 | 1.4 | 0 | 3 |
| CT-C-O | 67.4 | 123.2 |  | O-C-n-hn | 1 | 2.5 | 180 | -2 |
| CT-C-n | 66.8 | 115.18 |  | O-C-n-hn | 1 | 2 | 0 | 1 |
| CT-CT-H1 | 46.4 | 109.56 |  | O-C-CT-CT | 6 | 0 | 180 | 2 |
| CT-CT-ca | 63.1 | 112.07 |  | n-C-CT-CT | 1 | 0.1 | 0 | -4 |
| C-CT-H1 | 47 | 108.22 |  | n-C-CT-CT | 1 | 0.07 | 0 | 2 |
| C-CT-CT | 63.3 | 111.04 |  | CT-ca-ca-ha | 4 | 14.5 | 180 | 2 |
| C-n-hn | 48.3 | 117.55 |  | CT-ca-ca-ca | 4 | 14.5 | 180 | 2 |
| O-C-n | 74.2 | 123.05 |  | H1-CT-ca-ca | 6 | 0 | 0 | 2 |
| hn-n-hn | 39.6 | 117.95 |  | ca-ca-ca-ha | 4 | 14.5 | 180 | 2 |
| CT-ca-ca | 63.5 | 120.77 |  | ca-ca-ca-ca | 4 | 14.5 | 180 | 2 |
| H1-CT-H1 | 39.2 | 108.46 |  | ha-ca-ca-ha | 4 | 14.5 | 180 | 2 |
| H1-CT-ca | 47 | 109.56 |  |  |  |  |  |  |
| ca-ca-ha | 48.2 | 119.88 |  |  |  |  |  |  |
| ca-ca-ca | 66.6 | 120.02 |  |  | **NONBON** |  |  |  |
|  |  |  |  |  |  | N | 1.824 | 0.17 |
|  |  |  |  |  |  | H | 0.6 | 0.0157 |
|  |  |  |  |  |  | CT | 1.908 | 0.1094 |
|  |  |  |  |  |  | H1 | 1.387 | 0.0157 |
|  |  |  |  |  |  | C | 1.908 | 0.086 |
| **IMPROPER** |  |  |  |  |  | O | 1.6612 | 0.21 |
| CT-O-C-n | 10.5 | 180 | 2 |  |  | n | 1.824 | 0.17 |
| C-hn-n-hn | 1.1 | 180 | 2 |  |  | hn | 0.6 | 0.0157 |
| CT-ca-ca-ca | 1.1 | 180 | 2 |  |  | ca | 1.908 | 0.086 |
| ca-ca-ca-ha | 1.1 | 180 | 2 |  |  | ha | 1.459 | 0.015 |

**RC7**:

| **LABEL** | **TYPE** | **CHARGE** |  | **LABEL** | **TYPE** | **CHARGE** |  |  |
| --- | --- | --- | --- | --- | --- | --- | --- | --- |
| H25 | ha | 0.121137 |  | CB1 | cf | -0.101085 |  |  |
| CE2 | cd | -0.322833 |  | H32 | ha | 0.165187 |  |  |
| CZ | c | 0.571148 |  | CG1 | cd | -0.162216 |  |  |
| CE1 | cd | -0.322833 |  | ND1 | na | -0.314542 |  |  |
| H28 | ha | 0.121137 |  | H38 | hn | 0.303044 |  |  |
| CD1 | cc | -0.014484 |  | CE3 | cd | 0.371822 |  |  |
| H27 | ha | 0.119135 |  | H34 | h5 | 0.051158 |  |  |
| OH | o | -0.649362 |  | NE1 | nc | -0.676231 |  |  |
| CD2 | cc | -0.014484 |  | CD3 | cc | 0.292532 |  |  |
| H26 | ha | 0.119135 |  | H33 | h4 | 0.042148 |  |  |
| CG2 | cc | -0.244722 |  | C2 | cc | 0.22966 |  |  |
| CB2 | cf | 0.002202 |  | O2 | o | -0.414529 |  |  |
| H29 | ha | 0.120136 |  | N3 | n | -0.358492 |  |  |
| CA2 | cd | 0.133251 |  | CA3 | CT | -0.053639 |  |  |
| N2 | nd | -0.593326 |  | H35 | h1 | 0.075786 |  |  |
| C1 | cc | 0.412768 |  | H36 | h1 | 0.075786 |  |  |
| CA1 | ce | -0.185589 |  | C3 | C | 0.553528 |  |  |
| H31 | ha | 0.110125 |  | O3 | O | -0.562461 |  |  |
|  |  |  |  |  |  |  |  |  |
|  |  |  |  |  |  |  |  |  |
| **BOND** |  |  |  | **DIHE** |  |  |  |  |
| cd-ha | 349.1 | 1.084 |  | cd-c-cd-ha | 4 | 11.5 | 180 | 2 |
| c-cd | 371 | 1.468 |  | o-c-cd-ha | 4 | 11.5 | 180 | 2 |
| cc-cd | 500.9 | 1.373 |  | ha-cc-cd-ha | 4 | 16 | 180 | 2 |
| c-o | 637.7 | 1.218 |  | cc-cc-cd-ha | 4 | 16 | 180 | 2 |
| cc-ha | 349.1 | 1.084 |  | cd-c-cd-cc | 4 | 11.5 | 180 | 2 |
| cc-cc | 419.8 | 1.428 |  | cc-cc-cc-cd | 4 | 16 | 180 | 2 |
| cc-cf | 513 | 1.366 |  | cd-cc-cc-cf | 4 | 16 | 180 | 2 |
| cf-ha | 342.5 | 1.088 |  | ha-cc-cd-c | 4 | 16 | 180 | 2 |
| cd-cf | 386.9 | 1.454 |  | cc-cc-cd-c | 4 | 16 | 180 | 2 |
| cd-nd | 441.1 | 1.369 |  | cc-cc-cf-ha | 4 | 16 | 180 | 2 |
| cc-nd | 525.4 | 1.317 |  | cc-cc-cf-cd | 4 | 16 | 180 | 2 |
| cc-ce | 386.9 | 1.454 |  | cc-cc-cc-ha | 4 | 16 | 180 | 2 |
| cc-n | 425.1 | 1.381 |  | cf-cc-cc-ha | 4 | 16 | 180 | 2 |
| ce-ha | 342.5 | 1.088 |  | o-c-cd-cc | 4 | 11.5 | 180 | 2 |
| ce-cf | 538.6 | 1.351 |  | nd-cd-cf-cc | 4 | 4 | 180 | 2 |
| cd-na | 425.8 | 1.38 |  | cc-cd-cf-cc | 4 | 4 | 180 | 2 |
| hn-na | 408.4 | 1.01 |  | cf-cd-nd-cc | 2 | 9.5 | 180 | 2 |
| cd-h5 | 351.8 | 1.082 |  | o-cc-cd-cf | 4 | 16 | 180 | 2 |
| cd-nc | 525.4 | 1.317 |  | n-cc-cd-cf | 4 | 16 | 180 | 2 |
| cc-nc | 441.1 | 1.369 |  | nd-cd-cf-ha | 4 | 4 | 180 | 2 |
| cc-h4 | 352 | 1.082 |  | cc-cd-cf-ha | 4 | 4 | 180 | 2 |
| cc-o | 622.9 | 1.225 |  | ce-cc-nd-cd | 2 | 9.5 | 180 | 2 |
| CT-n | 328.7 | 1.462 |  | n-cc-nd-cd | 2 | 9.5 | 180 | 2 |
| CT-h1 | 330.6 | 1.097 |  | cd-cc-n-cc | 4 | 6.6 | 180 | 2 |
| C-CT | 313 | 1.524 |  | cd-cc-n-CT | 4 | 6.6 | 180 | 2 |
| C-O | 637.7 | 1.218 |  | o-cc-cd-nd | 4 | 16 | 180 | 2 |
|  |  |  |  | n-cc-cd-nd | 4 | 16 | 180 | 2 |
|  |  |  |  | nd-cc-ce-ha | 4 | 4 | 180 | 2 |
| **ANGLE** |  |  |  | nd-cc-ce-cf | 4 | 4 | 180 | 2 |
| c-cd-ha | 46.9 | 116.64 |  | nd-cc-n-cc | 4 | 6.6 | 180 | 2 |
| cc-cd-ha | 48.5 | 121.76 |  | nd-cc-n-CT | 4 | 6.6 | 180 | 2 |
| cd-c-cd | 64.6 | 115.84 |  | cc-ce-cf-ha | 4 | 26.6 | 180 | 2 |
| cd-c-o | 69.1 | 123.93 |  | cc-ce-cf-cd | 4 | 26.6 | 180 | 2 |
| cd-cc-ha | 48.5 | 121.76 |  | h1-CT-n-cc | 6 | 0 | 0 | 2 |
| cc-cc-cd | 68.2 | 114.19 |  | C-CT-n-cc | 6 | 0 | 0 | 2 |
| c-cd-cc | 65.1 | 121.35 |  | ce-cc-n-cc | 4 | 6.6 | 180 | 2 |
| cc-cc-cc | 67.9 | 110.7 |  | ce-cc-n-CT | 4 | 6.6 | 180 | 2 |
| cc-cc-cf | 65.9 | 122.72 |  | na-cd-cf-ce | 4 | 4 | 180 | 2 |
| cc-cc-ha | 47.1 | 121.07 |  | cc-cd-cf-ce | 4 | 4 | 180 | 2 |
| cc-cf-ha | 50.1 | 114.95 |  | ha-ce-cf-ha | 4 | 26.6 | 180 | 2 |
| cc-cf-cd | 63.2 | 130.61 |  | ha-ce-cf-cd | 4 | 26.6 | 180 | 2 |
| cf-cd-nd | 67.2 | 121.1 |  | cf-cd-na-hn | 4 | 6.8 | 180 | 2 |
| cc-cd-cf | 63.7 | 128.05 |  | cf-cd-na-cd | 4 | 6.8 | 180 | 2 |
| cd-cf-ha | 47.5 | 115.44 |  | nc-cc-cd-cf | 4 | 16 | 180 | 2 |
| cc-nd-cd | 71.8 | 105.49 |  | h4-cc-cd-cf | 4 | 16 | 180 | 2 |
| cd-cc-o | 17.8 | 136.06 |  | na-cd-cf-ha | 4 | 4 | 180 | 2 |
| cd-cc-n | 68.9 | 121.33 |  | h5-cd-na-cd | 4 | 6.8 | 180 | 2 |
| cc-cd-nd | 72.2 | 111.65 |  | nc-cd-na-cd | 4 | 6.8 | 180 | 2 |
| ce-cc-nd | 68.1 | 121.7 |  | nc-cc-cd-na | 4 | 16 | 180 | 2 |
| n-cc-nd | 71.5 | 123 |  | h4-cc-cd-na | 4 | 16 | 180 | 2 |
| cc-ce-ha | 47.5 | 115.44 |  | na-cd-nc-cc | 2 | 9.5 | 180 | 2 |
| cc-ce-cf | 64.6 | 126.14 |  | h5-cd-na-hn | 4 | 6.8 | 180 | 2 |
| cc-n-cc | 68.8 | 108.92 |  | nc-cd-na-hn | 4 | 6.8 | 180 | 2 |
| CT-n-cc | 63.3 | 120.85 |  | cd-cc-nc-cd | 2 | 9.5 | 180 | 2 |
| ce-cc-n | 68.3 | 124.2 |  | h4-cc-nc-cd | 2 | 9.5 | 180 | 2 |
| ce-cf-ha | 49.8 | 118.22 |  | h5-cd-nc-cc | 2 | 9.5 | 180 | 2 |
| cd-cf-ce | 64.6 | 126.14 |  | cc-cd-na-hn | 4 | 6.8 | 180 | 2 |
| cf-ce-ha | 49.8 | 118.22 |  | cc-cd-na-cd | 4 | 6.8 | 180 | 2 |
| cf-cd-na | 66.1 | 124.35 |  | cc-cd-nd-cc | 2 | 9.5 | 180 | 2 |
| cd-na-hn | 46.8 | 125.5 |  | o-cc-n-cc | 4 | 6.6 | 180 | 2 |
| cd-na-cd | 68.5 | 109.9 |  | o-cc-n-CT | 4 | 6.6 | 180 | 2 |
| cd-cc-nc | 72.2 | 111.65 |  | n-cc-ce-ha | 4 | 4 | 180 | 2 |
| cd-cc-h4 | 47.3 | 128.48 |  | n-cc-ce-cf | 4 | 4 | 180 | 2 |
| cc-cd-na | 73.4 | 106.99 |  | O-C-CT-n | 6 | 0 | 180 | 2 |
| h5-cd-na | 49.6 | 121.55 |  | O-C-CT-h1 | 1 | 0.8 | 0 | -1 |
| na-cd-nc | 74.9 | 112.22 |  | O-C-CT-h1 | 1 | 0 | 0 | -2 |
| cc-nc-cd | 71.8 | 105.49 |  | O-C-CT-h1 | 1 | 0.08 | 180 | 3 |
| h5-cd-nc | 50.6 | 125.52 |  |  |  |  |  |  |
| h4-cc-nc | 50 | 121.14 |  |  |  |  |  |  |
| n-cc-o | 73.357 | 123.9 |  |  |  |  |  |  |
| h1-CT-n | 49.8 | 108.88 |  |  |  |  |  |  |
| C-CT-n | 67 | 109.06 |  |  |  |  |  |  |
| CT-C-O | 67.4 | 123.2 |  |  |  |  |  |  |
| h1-CT-h1 | 39.2 | 108.46 |  |  |  |  |  |  |
| C-CT-h1 | 47 | 108.22 |  |  | **NONBON** |  |  |  |
|  |  |  |  |  |  | ha | 1.459 | 0.015 |
|  |  |  |  |  |  | cd | 1.908 | 0.086 |
| **IMPROPER** |  |  |  |  |  | c | 1.908 | 0.086 |
| c-cc-cd-ha | 1.1 | 180 | 2 |  |  | cc | 1.908 | 0.086 |
| cd-cd-c-o | 10.5 | 180 | 2 |  |  | o | 1.6612 | 0.21 |
| cc-cd-cc-ha | 1.1 | 180 | 2 |  |  | cf | 1.908 | 0.086 |
| cc-cc-cc-cf | 1.1 | 180 | 2 |  |  | nd | 1.824 | 0.17 |
| cc-cd-cf-ha | 1.1 | 180 | 2 |  |  | ce | 1.908 | 0.086 |
| cc-cf-cd-nd | 1.1 | 180 | 2 |  |  | na | 1.824 | 0.17 |
| ce-n-cc-nd | 1.1 | 180 | 2 |  |  | hn | 0.6 | 0.0157 |
| cc-cf-ce-ha | 1.1 | 180 | 2 |  |  | h5 | 1.359 | 0.015 |
| cd-ce-cf-ha | 1.1 | 180 | 2 |  |  | nc | 1.824 | 0.17 |
| cc-cf-cd-na | 1.1 | 180 | 2 |  |  | h4 | 1.409 | 0.015 |
| cd-cd-na-hn | 1.1 | 180 | 2 |  |  | n | 1.824 | 0.17 |
| h5-na-cd-nc | 1.1 | 180 | 2 |  |  | CT | 1.908 | 0.1094 |
| cd-h4-cc-nc | 1.1 | 180 | 2 |  |  | h1 | 1.387 | 0.0157 |
| cd-n-cc-o | 1.1 | 180 | 2 |  |  | C | 1.908 | 0.086 |
| CT-cc-n-cc | 1.1 | 180 | 2 |  |  | O | 1.6612 | 0.21 |


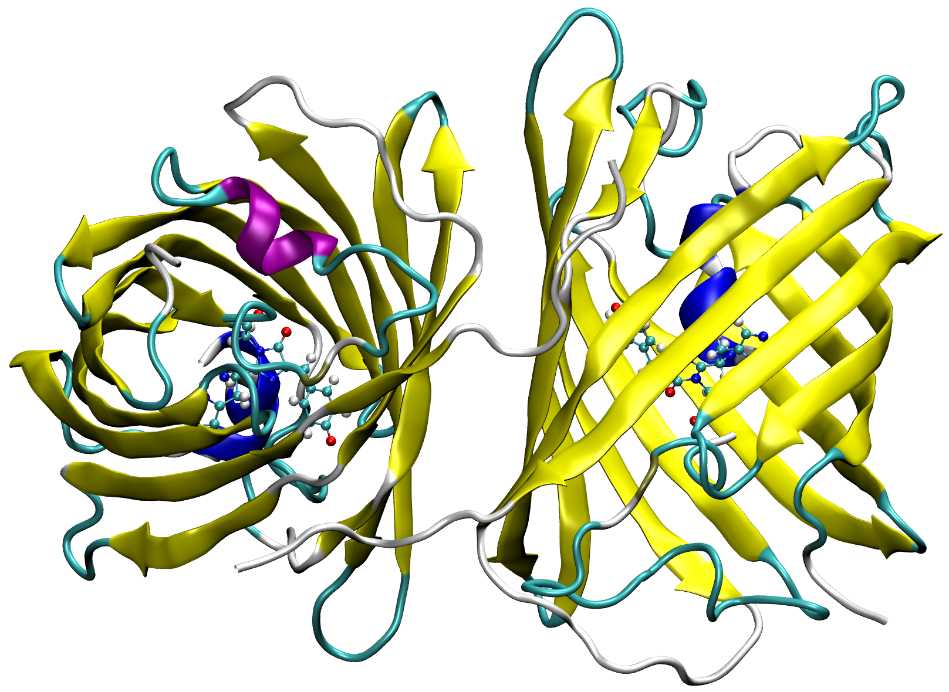


**Figure S10**. Snapshot of the last geometry for the green QuetzalFP obtained from the classical 1 μs MD.

| 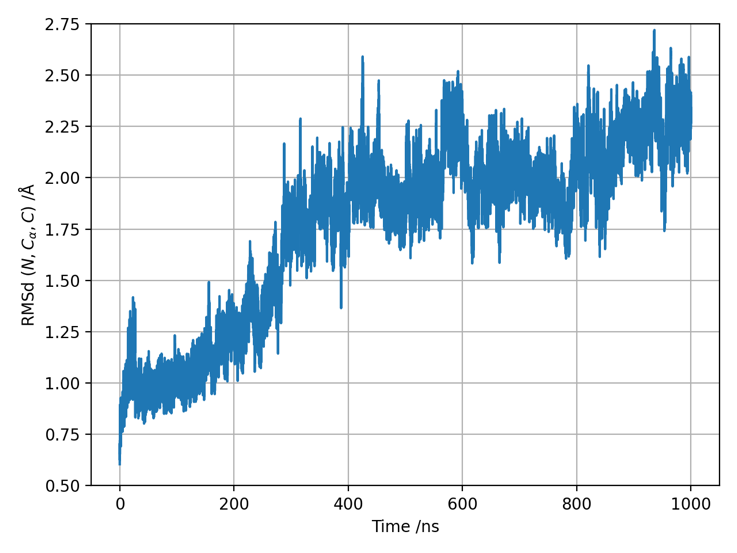 | 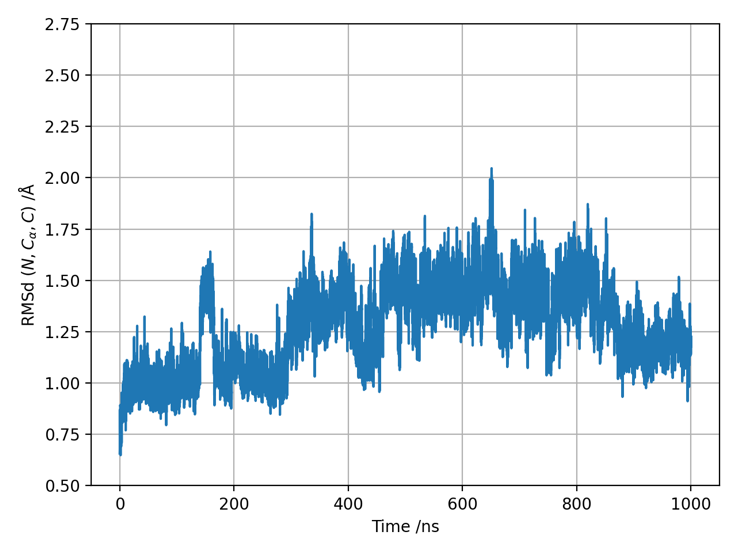 |
| --- | --- |
| 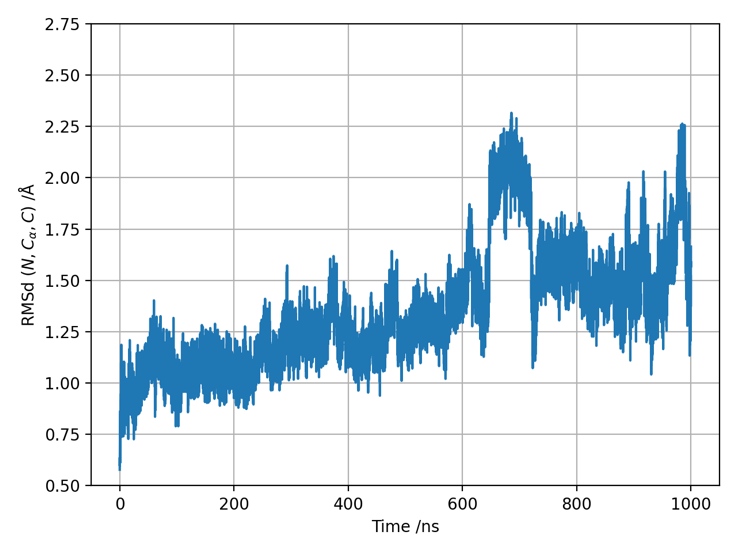 | 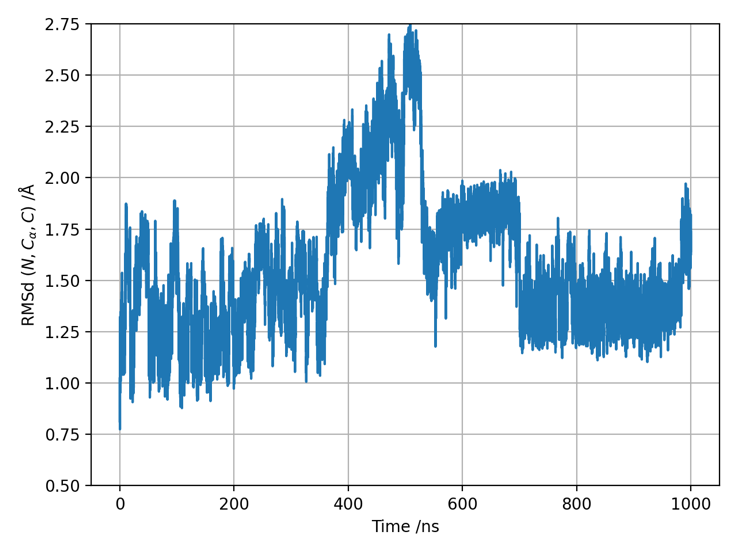 |

**Figure S11**. Root mean square deviation (RMSd, in Å) calculated at the backbone atoms for the classical 1 μs MD for the green (upper panel) and red (lower panel) QuetzalFP and EosFP (from left to right).

| 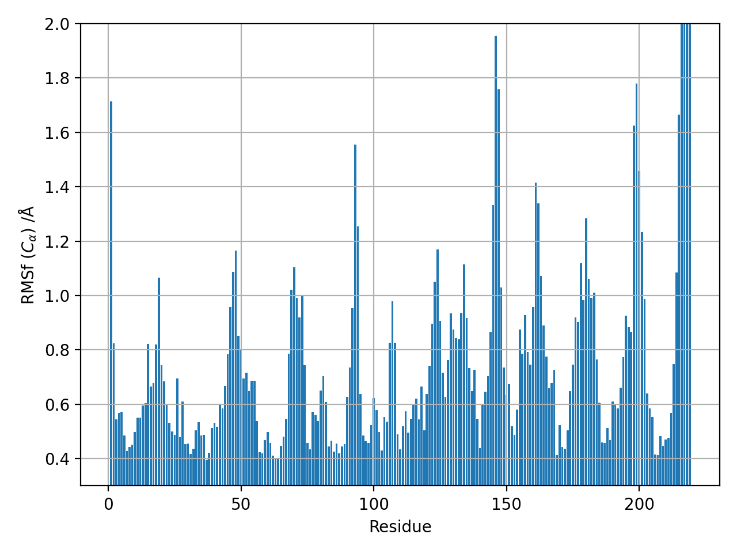 | 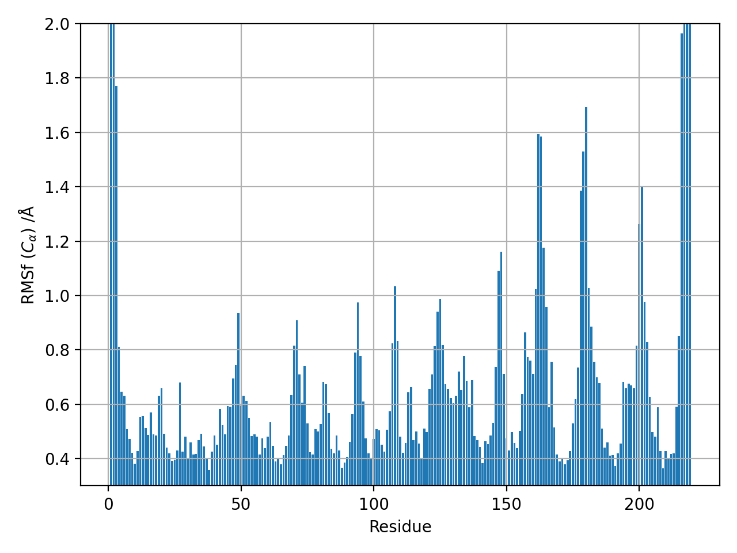 |
| --- | --- |
| 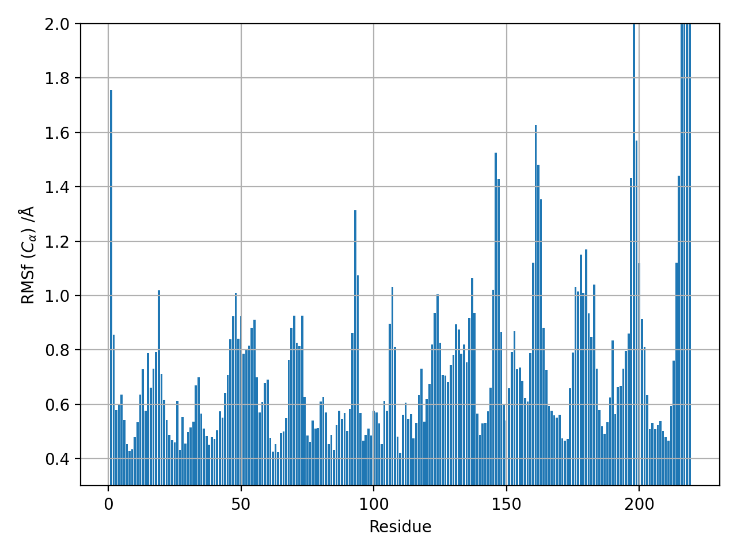 | 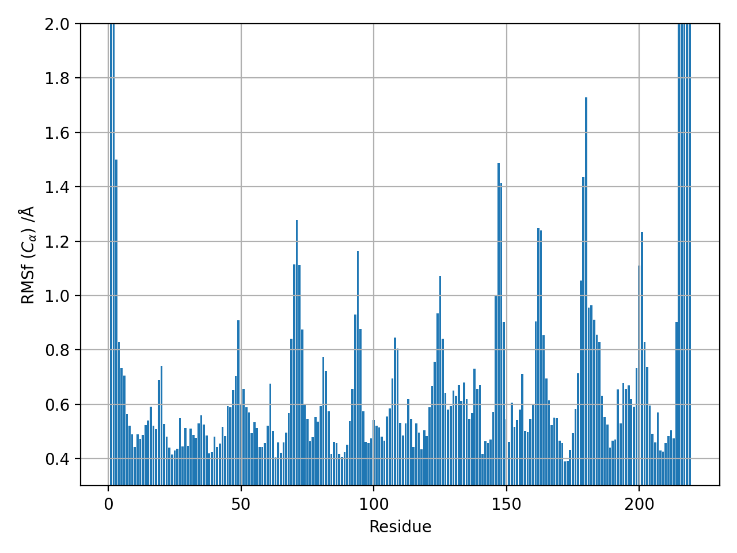 |

**Figure S12**. Root mean square fluctuation (RMSf, in Å) calculated at the C_α_ atoms for the classical 1 μs MD for the green (upper panel) and red (lower panel) QuetzalFP and EosFP (from left to right).

| 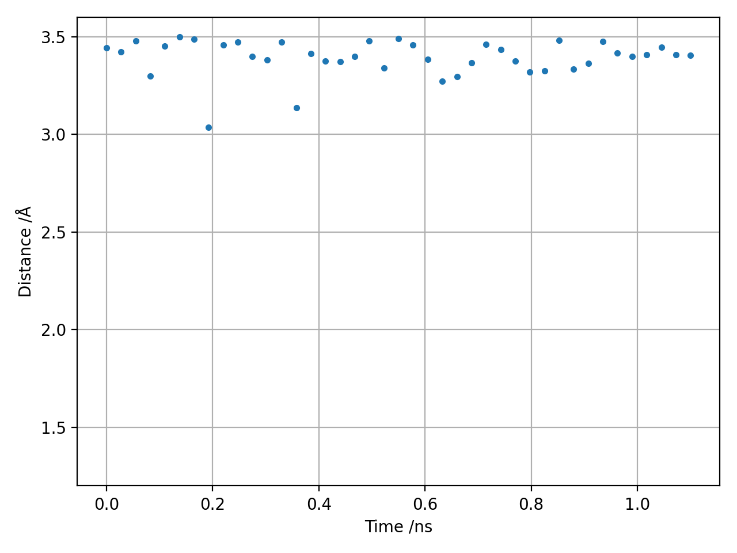 | 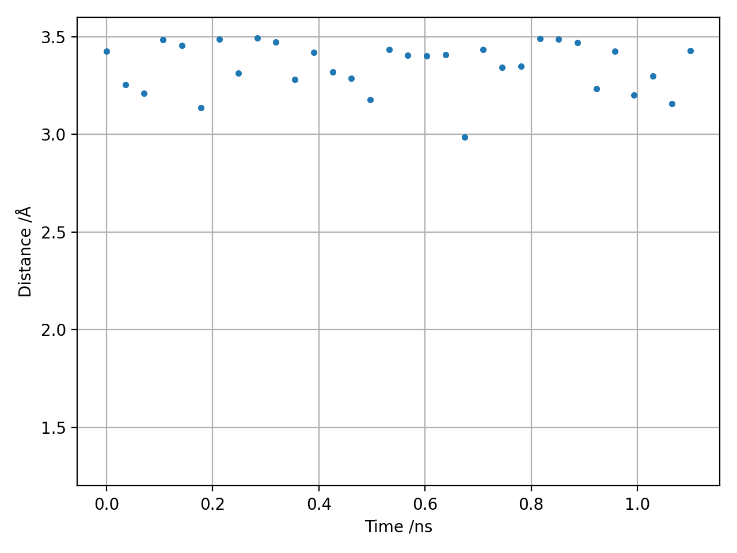 |
| --- | --- |
| 3.39 ± 0.09 Å [0.004] | 3.35 ± 0.13 Å [0.002] |
| 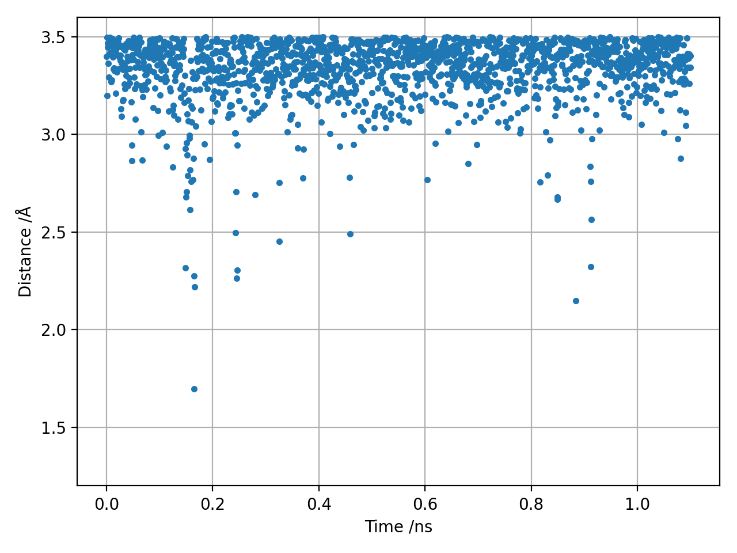 | 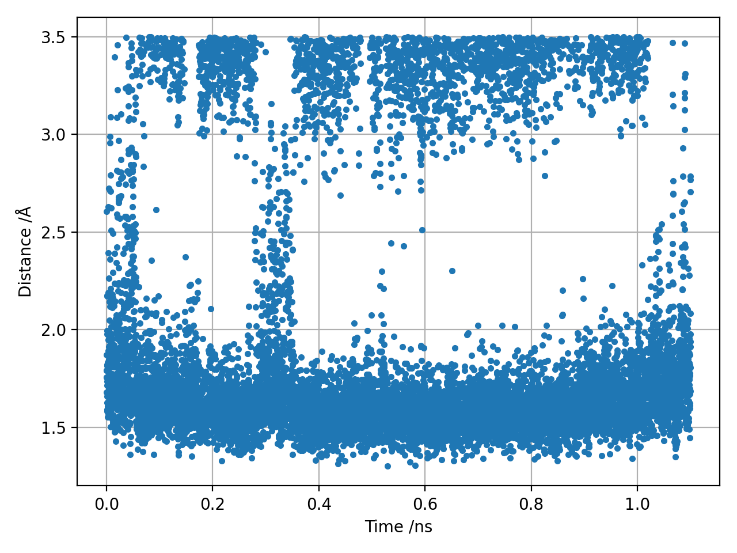 |
| 3.33 ± 0.17 Å [0.15] | 1.90 ± 0.70 Å [1.04] |

**Figure S13**. Hydrogen bond interactions between the carbonylic oxygen moiety of the imidazole ring of the chromophore and the water molecules of the solvent for the green (upper panel) and red (lower panel) QuetzalFP and EosFP (from left to right). The average distance values (in Å), standard deviations and residence times (expressed as per-unit, in brackets) are also indicated.

| 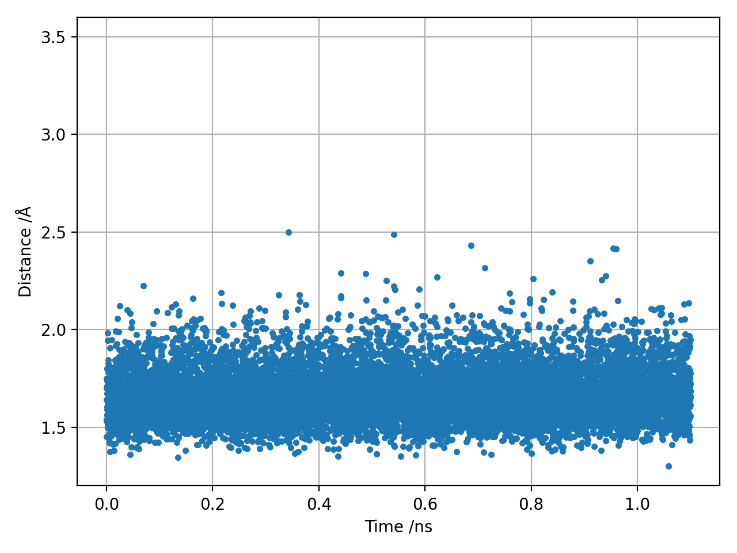 |  | 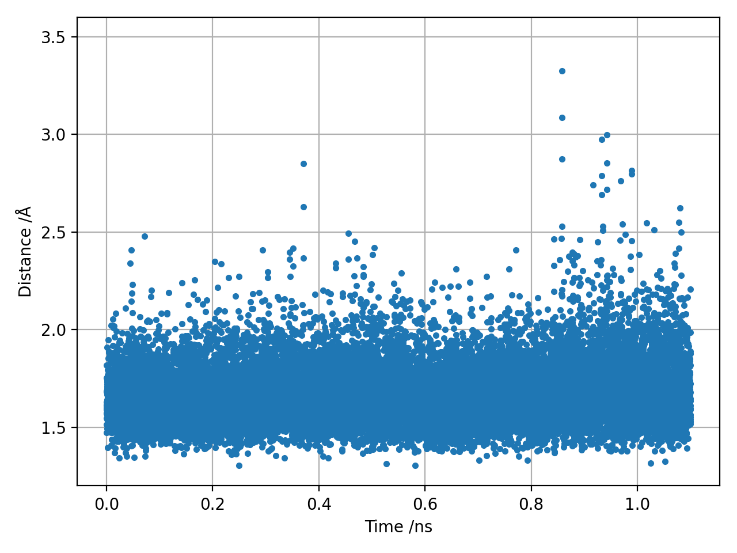 |
| --- | --- | --- |
| 1.67 ± 0.13 Å [1.000] |  | 1.67 ± 0.15 Å [1.999] |
| 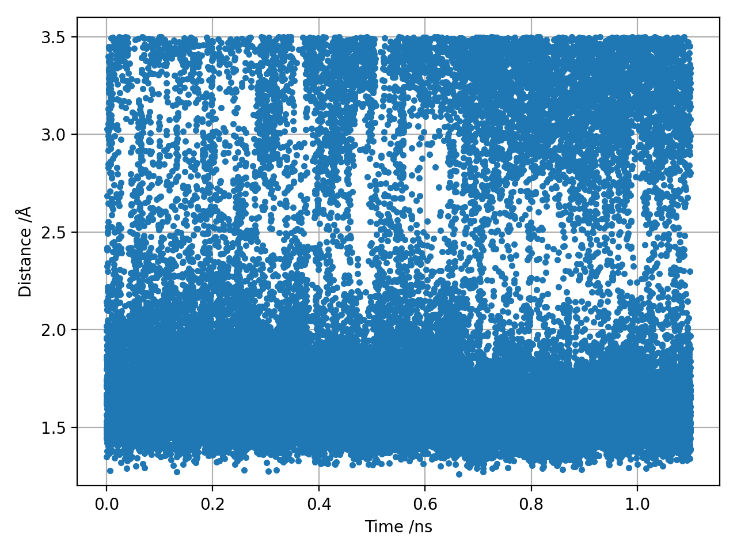 |  | 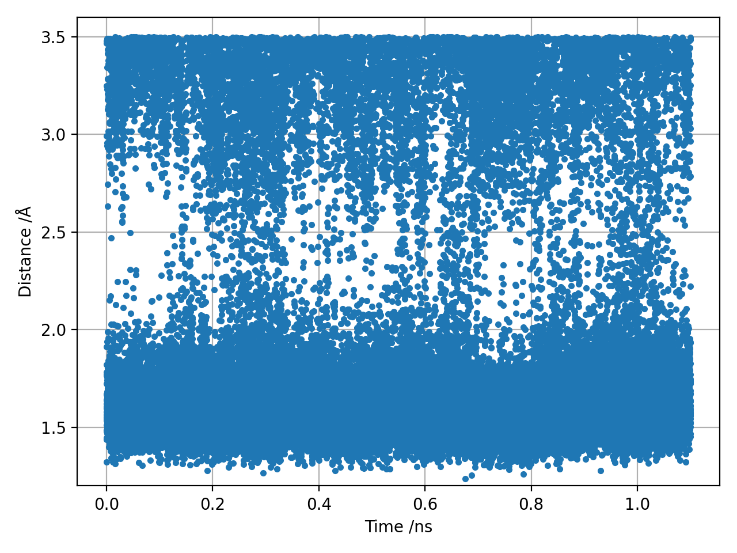 |
| 1.90 ± 0.58 Å [4.434] |  | 1.92 ± 0.6 Å [4.737] |

**Figure S14**. Hydrogen bond interactions between the phenolic oxygen moiety of the chromophore and the water molecules of the solvent of the green (upper panel) and red (lower panel) QuetzalFP and EosFP (from left to right). The average distance values (in Å), standard deviations and the residence time (expressed as per- unit, in brackets) are also indicated.

| 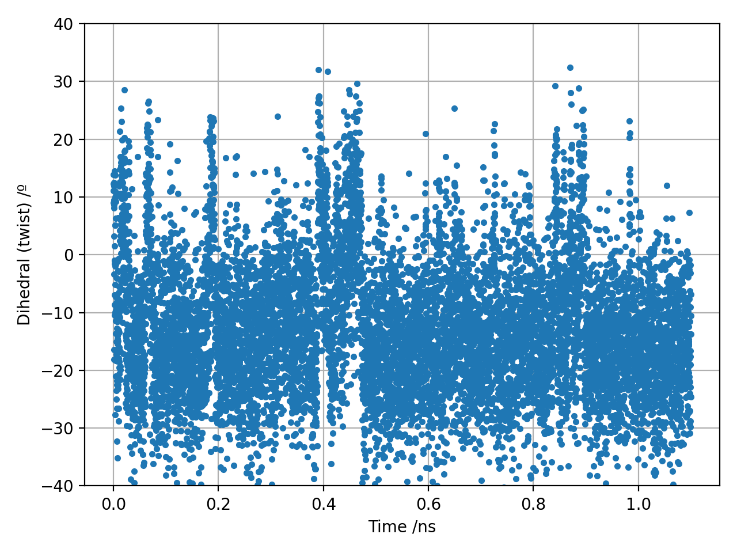 | 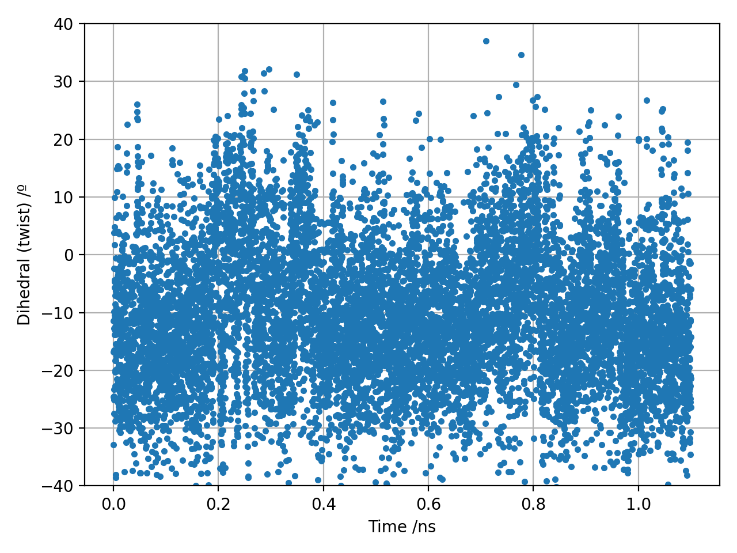 |
| --- | --- |
| -13 ± 12 ° | -10 ± 12 ° |
| 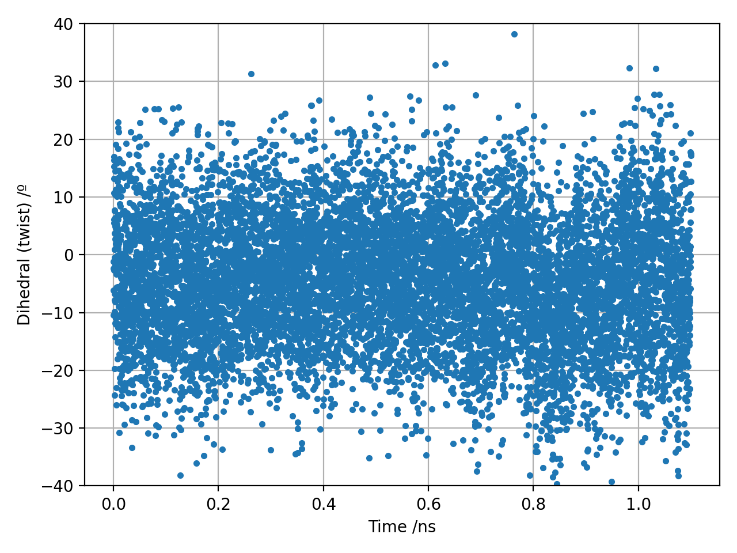 | 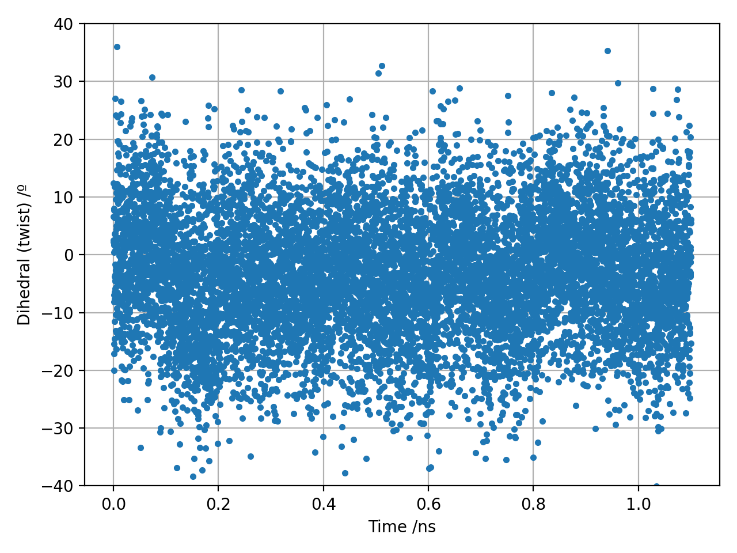 |
| -5 ± 11 ° | -3 ± 11 ° |


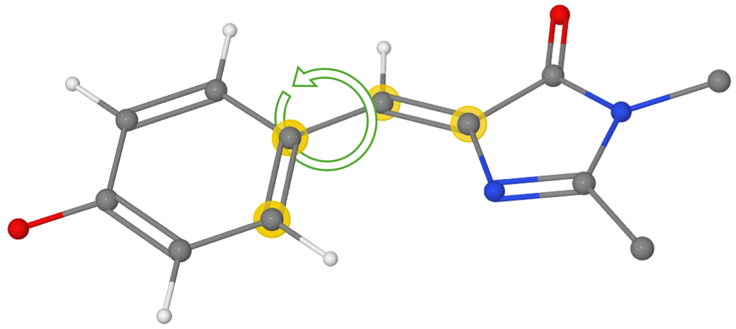


**Figure S15**. Evolution of the P dihedral angle in the chromophore (see picture below) of the green (upper panel) and red (lower panel) QuetzalFP and EosFP (from left to right). The average values (in degrees) and the standard deviations are also indicated.

| 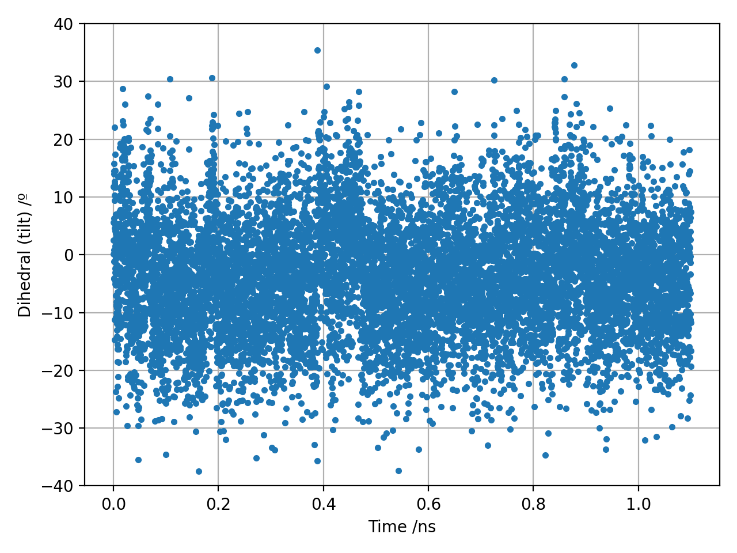 | 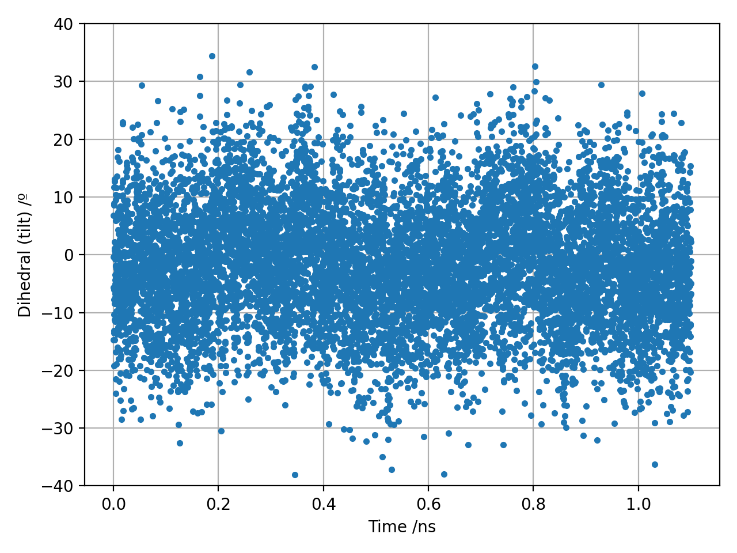 |
| --- | --- |
| -4 ± 10 ° | -2 ± 10 ° |
| 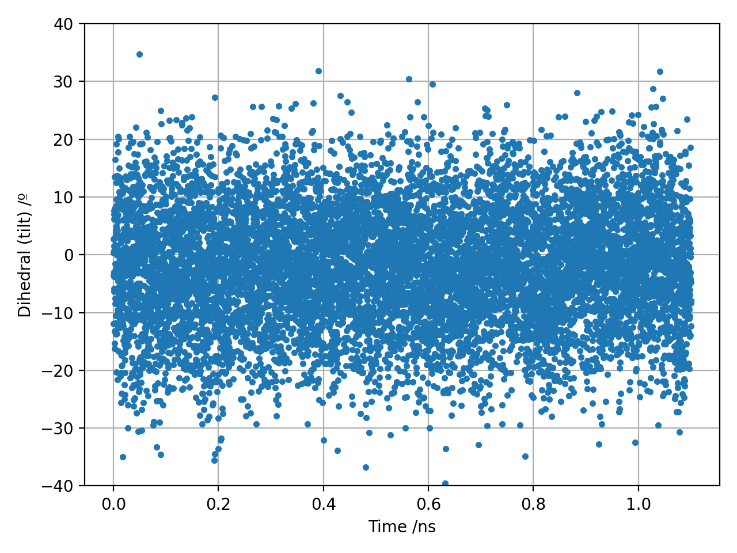 | 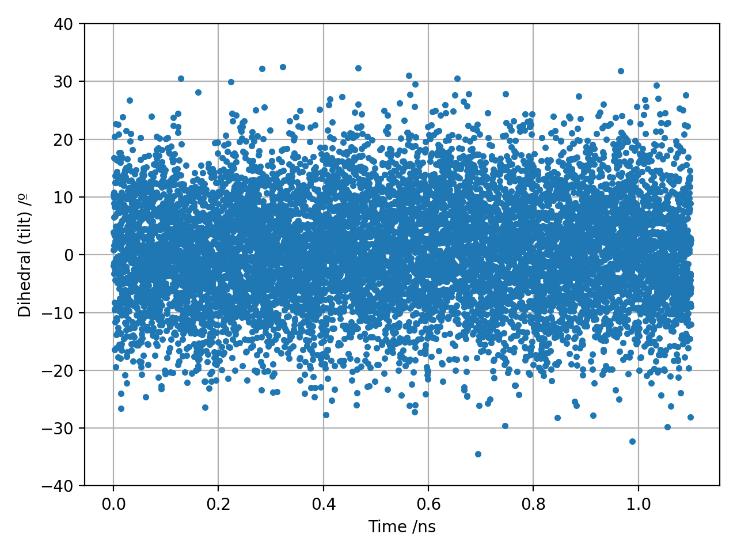 |
| -2 ± 10 ° | 1 ± 9 ° |


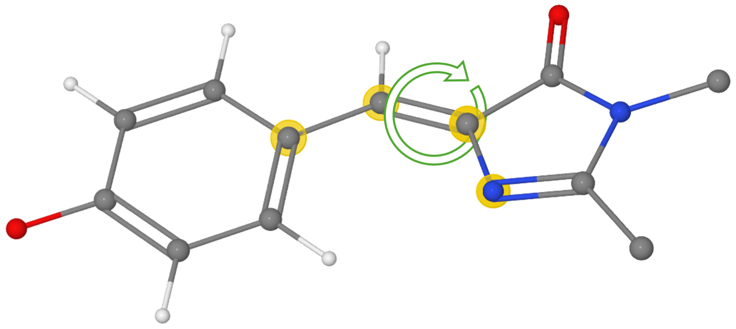


**Figure S16**. Evolution of the I dihedral angle in the chromophore (see picture below) of the green (upper panel) and red (lower panel) QuetzalFP and EosFP (from left to right). The average values (in degrees) and the standard deviations are also indicated.

| 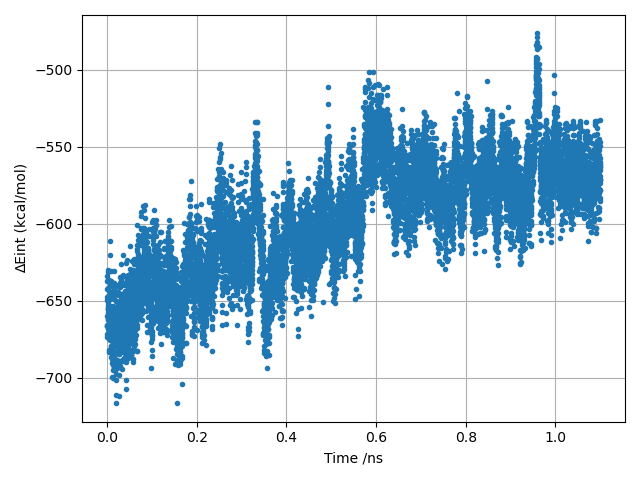 | 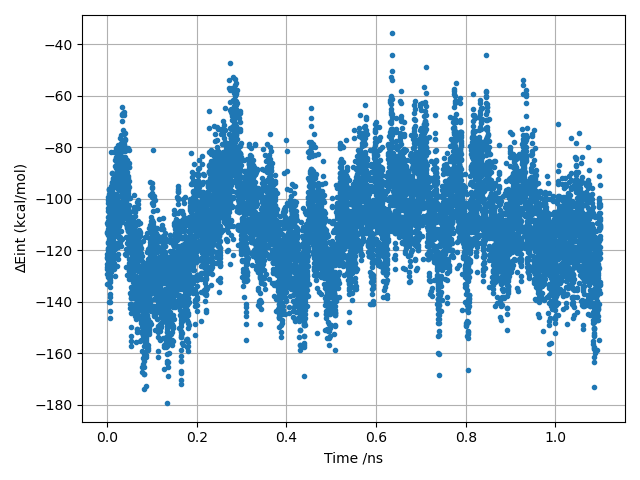 |
| --- | --- |
| -597 ± 38 kcal/mol | -111 ± 19 kcal/mol |
| 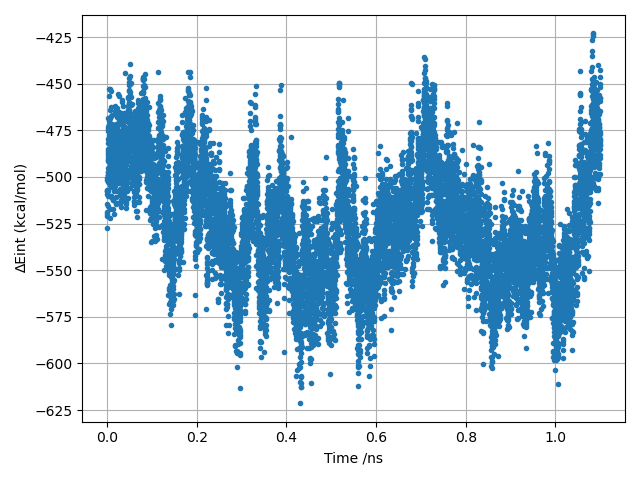 | 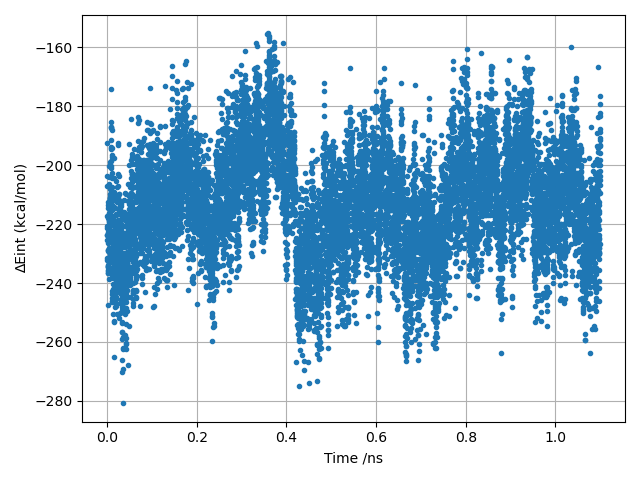 |
| -525 ± 31 kcal/mol | -213 ± 18 kcal/mol |

*These results have been obtained by averaging total energy differences as follows:*

$${\Delta E}_{int}=\left\langle E_{dimer}-E_{monomerA}-E_{monomerB} \right\rangle$$

*where water molecules and counterions have been completely removed in each model, and the periodic boundary conditions (PBC) have been substituted by a cutoff radius of 999 Å.*

**Figure S17**. Evolution of the interaction energy between both subunits (in kcal/mol) for the green (upper panel) and red (lower panel) QuetzalFP and EosFP (from left to right). The average values and the standard deviations are also indicated.

| 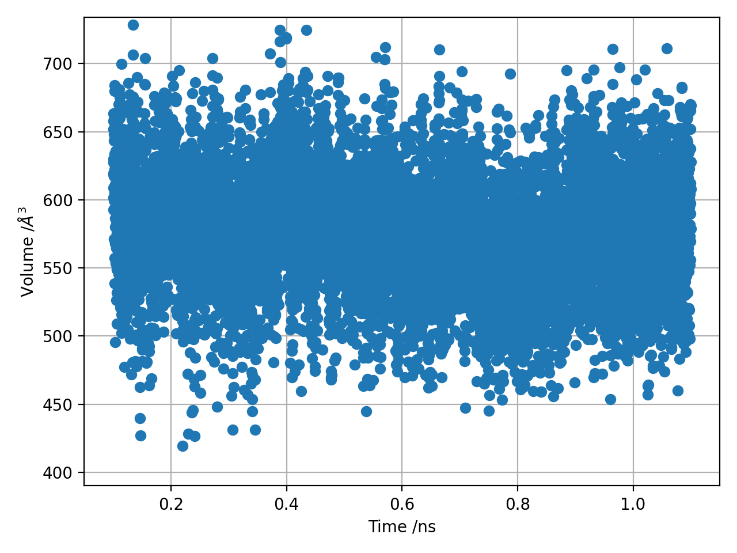 | 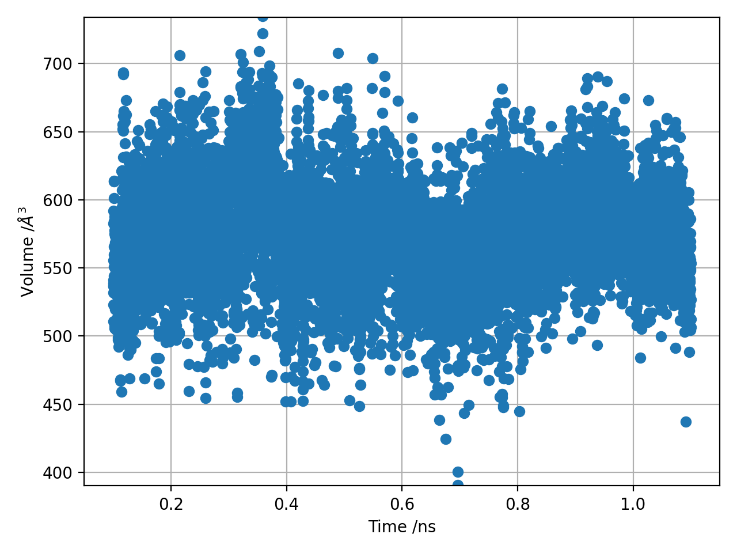 |
| --- | --- |
| 575 ± 43 Å^3^ | 574 ± 37 Å^3^ |
| 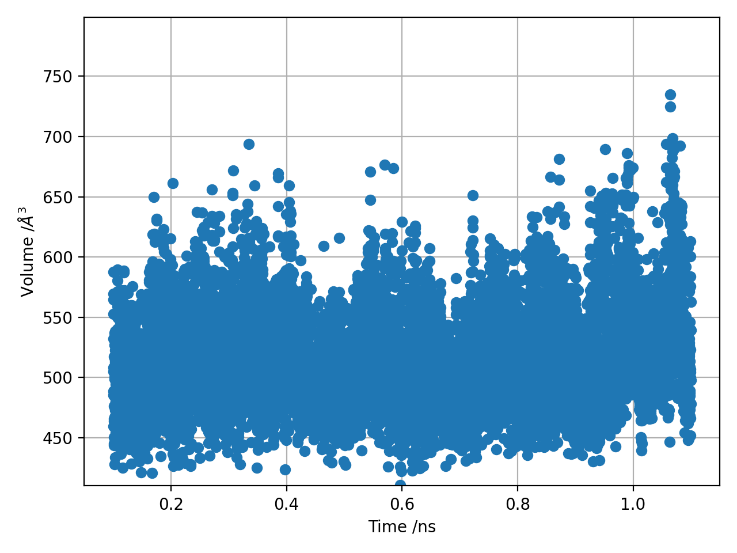 | 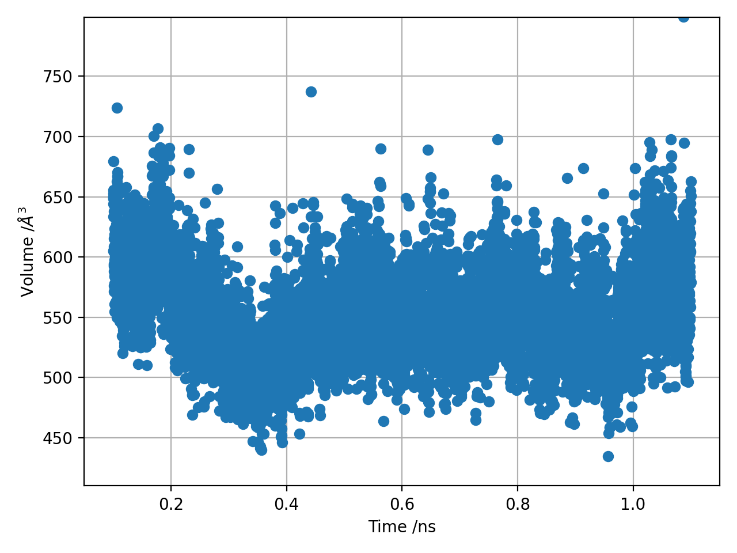 |
| 516 ± 41 Å^3^ | 552 ± 38 Å^3^ |

**Figure S18**. Volume (in Å^3^) of the chromophore binding pocket for the last 1 ns of QM/MM/MD simulations for the green (upper panel) and red (lower panel) QuetzalFP and EosFP (from left to right). The average values (in Å^3^) and the standard deviations are also indicated.


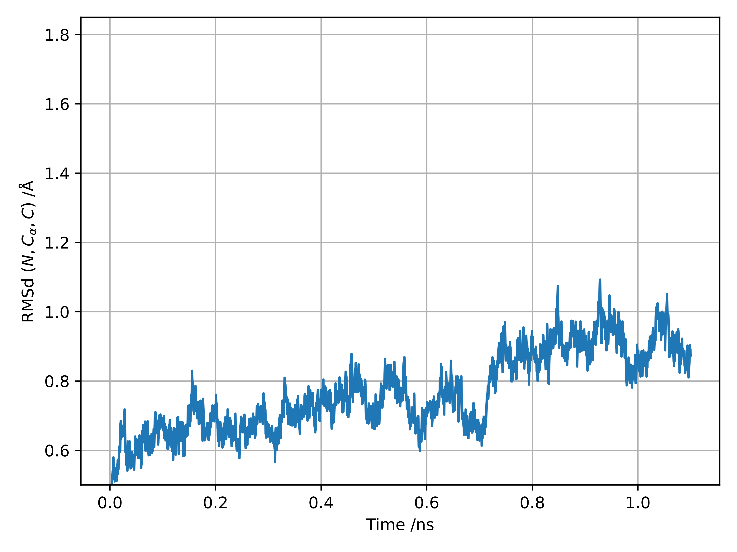

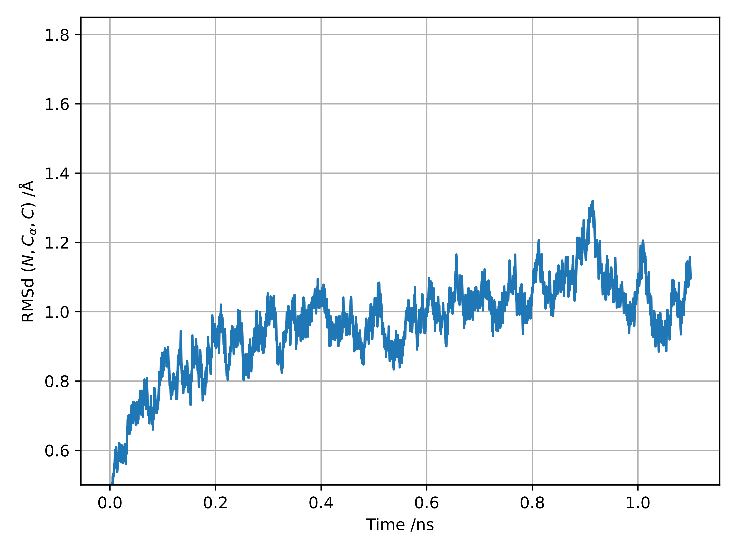

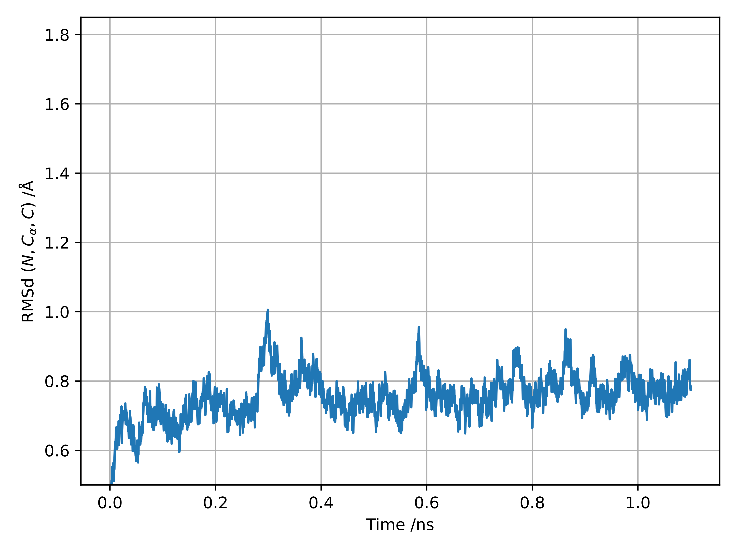


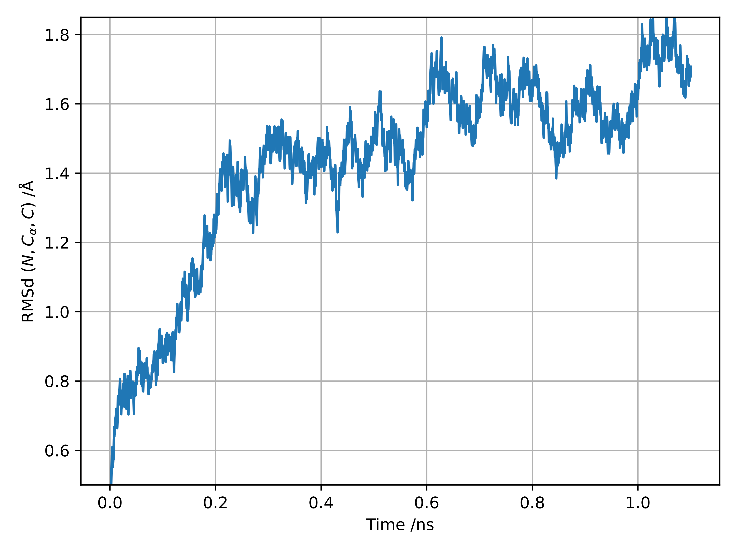


**Figure S19**. Root mean square deviation (RMSd, in Å) calculated at the backbone atoms

for the QM/MM/MD 1.1 ns MD for the green (upper panel) and red (lower panel) QuetzalFP and EosFP (from left to right).

| 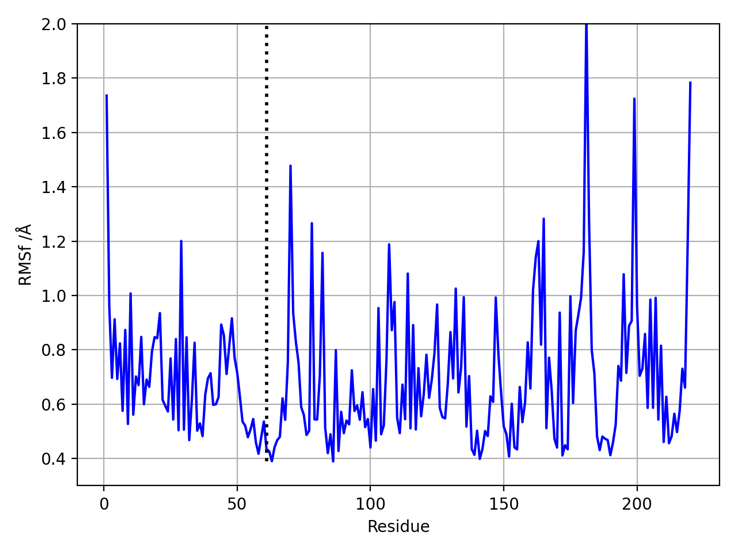 | 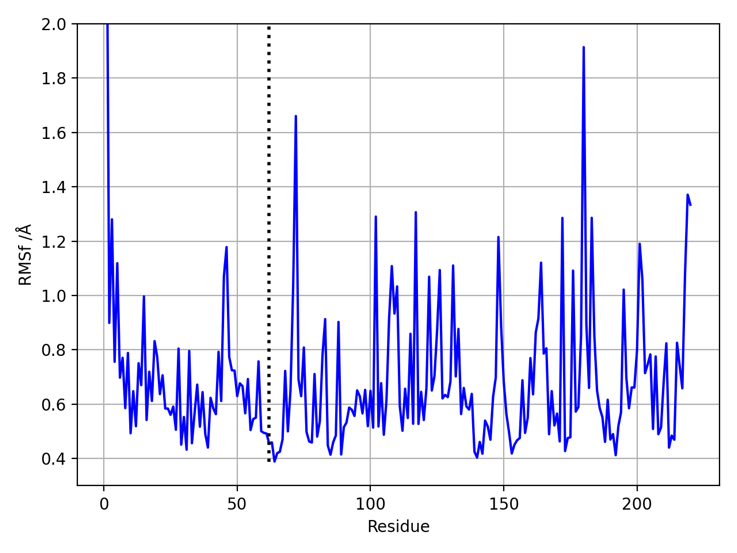 |
| --- | --- |
| 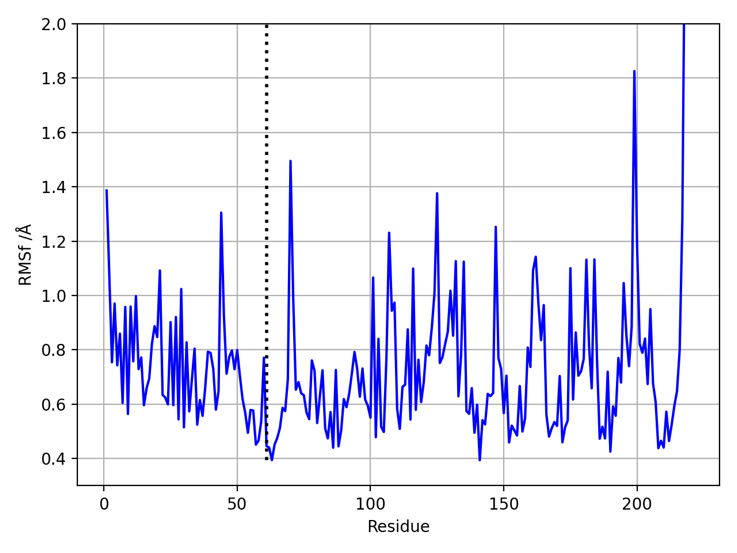 | 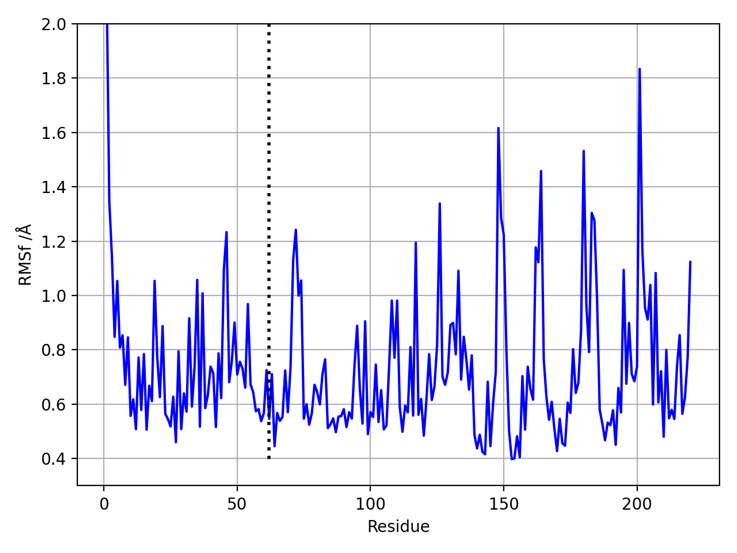 |

**Figure S20**. Root mean square fluctuation (RMSf, in Å) calculated for the QM/MM/MD 1.1 ns MD at the C_α_ atoms for the green (upper panel) and red (lower panel) QuetzalFP and EosFP (from left to right). The chromophore is highlighted with a dotted line.

**Table S3**. Main interactions between the residues in both green QuetzalFP (upper panel) and green EosFP (lower panel) chromophore binding pockets. Distances expressed in Å, and residence times expressed as per- unit. A representative snapshot of each active site is also depicted for each system (the chromophore depicted in ball and sticks).

| Residue @ Atom | Residue @ Atom | Distance (Å) | Residence time  (per unit) |
| --- | --- | --- | --- |
| CR8_66@O2 | ARG_69@HH12 | 2.830 | 1.000 |
| CR8_66@O2 | ARG_69@HH22 | 2.959 | 0.984 |
| CR8_66@O2 | ARG_94@HH12 | 3.315 | 0.594 |
| CR8_66@O2 | ARG_94@HH22 | 2.811 | 1.000 |
| GLU_147@OE1 | HIP_196@HE2 | 2.788 | 1.000 |
| GLU_214@OE2 | HIP_196@HD1 | 2.887 | 0.933 |
| GLU_214@OE1 | HIP_196@HD1 | 3.151 | 0.768 |
| GLU_214@OE2 | GLN_41@HE22 | 2.912 | 0.066 |
| GLN_41@O | GLU_214@H | 2.925 | 0.998 |

| Residue @ Atom | Residue @ Atom | Distance (Å) | Residence time  (per unit) |
| --- | --- | --- | --- |
| CR8_64@O2 | ARG_66@HH12 | 2.826 | 1.000 |
| CR8_64@O2 | ARG_66@HH22 | 2.948 | 0.991 |
| CR8_64@O2 | ARG_91@HH22 | 2.827 | 0.998 |
| CR8_64@O2 | ARG_91@HH12 | 3.171 | 0.892 |
| GLU_144@OE1 | HIP_194@HE2 | 2.760 | 1.000 |
| GLU_212@OE2 | HIP_194@HD1 | 3.081 | 0.756 |
| GLU_212@OE1 | HIP_194@HD1 | 2.853 | 0.732 |
| GLU_212@OE1 | GLN_38@HE22 | 2.843 | 0.268 |
| GLN_38@O | GLU_212@H | 2.877 | 0.997 |

| QuetzalFP | EosFP |
| --- | --- |
| 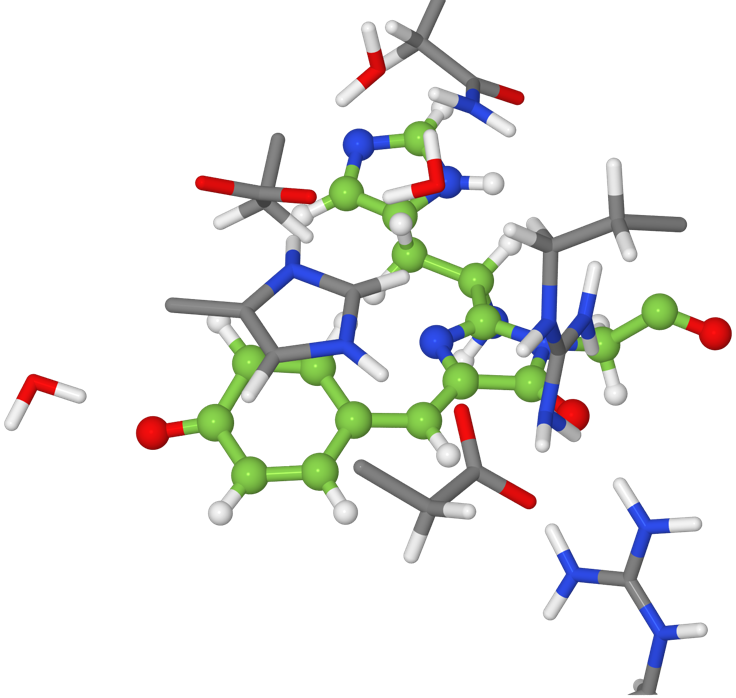 | 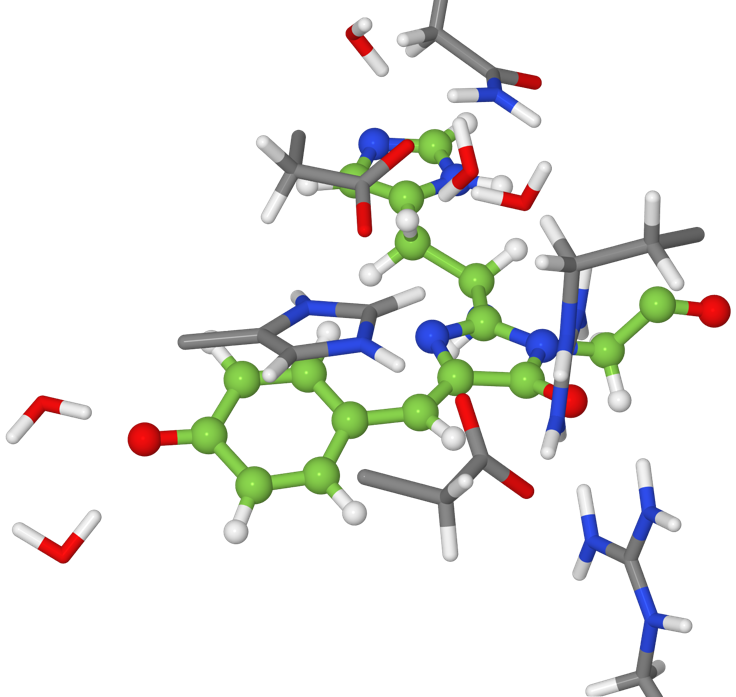 |


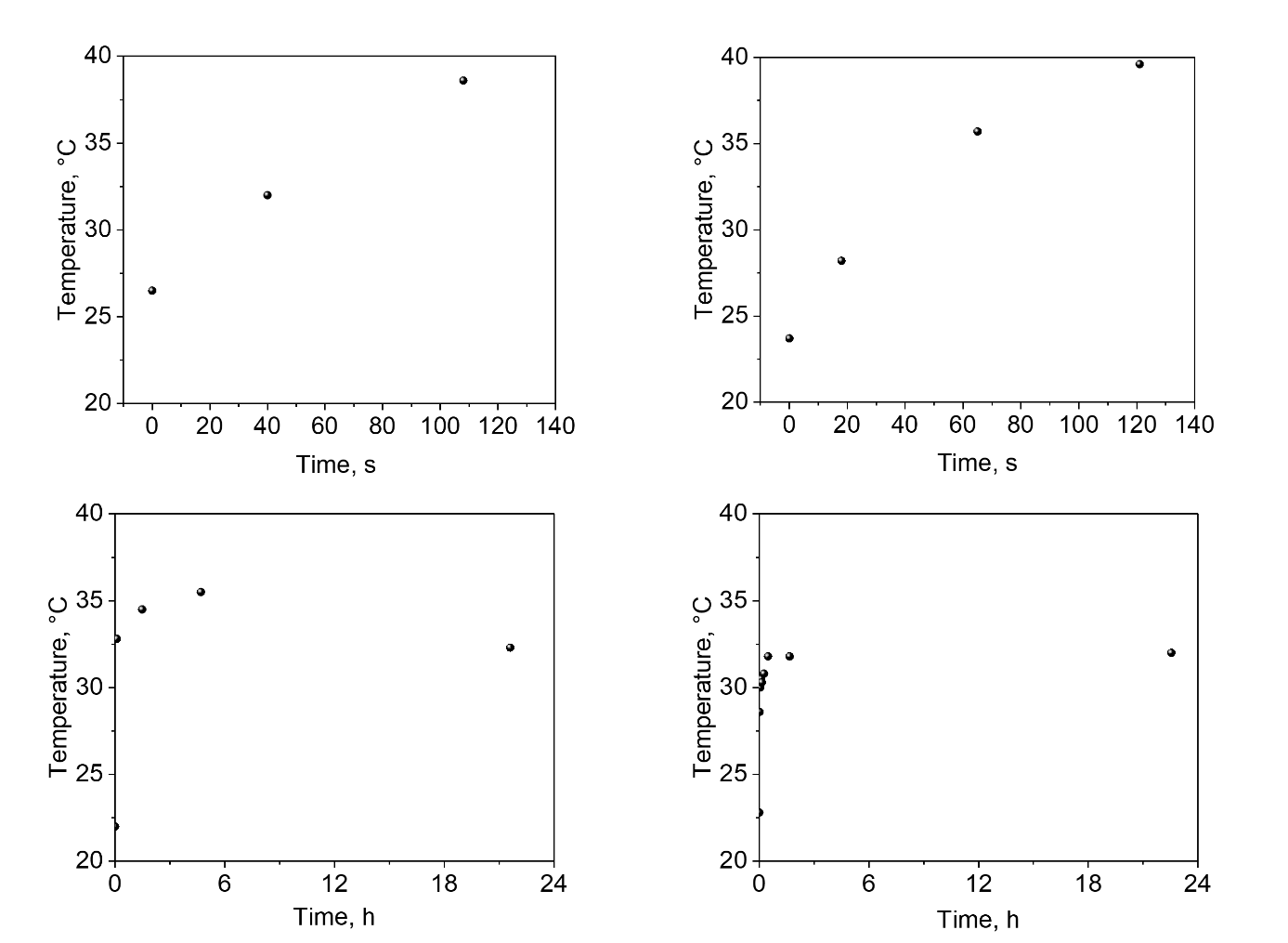


**Figure S21.** Heating behavior of the devices with green (top) and red (bottom) QuetzalFP (left) and EosFP (right) HPC coatings.

**References**

[1] R. Ando, H. Mizuno, A. Miyawaki, *Science* **2004**, *306*, 1370.

[2] N. V. Pletneva, V. Z. Pletnev, K. A. Lukyanov, N. G. Gurskaya, E. A. Goryacheva, V. I. Martynov, A. Wlodawer, Z. Dauter, S. Pletnev, *J. Biol. Chem.* **2010**, *285*, 15978.

[3] M. V. Matz, A. F. Fradkov, Y. A. Labas, A. P. Savitsky, A. G. Zaraisky, M. L. Markelov, S. A. Lukyanov, *Nat. Biotechnol.* **1999**, *17*, 969.

[4] D. A. Shagin, E. V. Barsova, Y. G. Yanushevich, A. F. Fradkov, K. A. Lukyanov, Y. A. Labas, T. N. Semenova, J. A. Ugalde, A. Meyers, J. M. Nunez, E. A. Widder, S. A. Lukyanov, M. V. Matz, *Mol. Biol. Evol.* **2004**, *21*, 841.

[5] D. C. Prasher, V. K. Eckenrode, W. W. Ward, F. G. Prendergast, M. J. Cormier, *Gene* **1992**, *111*, 229.

[6] G. G. Lambert, H. Depernet, G. Gotthard, D. T. Schultz, I. Navizet, T. Lambert, S. R. Adams, A. Torreblanca-Zanca, M. Chu, D. S. Bindels, V. Levesque, J. N. Moffatt, A. Salih, A. Royant, N. C. Shaner, *PLoS Biol.* **2020**, *18*, e3000936.

[7] H. Masuda, Y. Takenaka, A. Yamaguchi, S. Nishikawa, H. Mizuno, *Gene* **2006**, *372*, 18.

[8] Y. A. Labas, N. G. Gurskaya, Y. G. Yanushevich, A. F. Fradkov, K. A. Lukyanov, S. A. Lukyanov, M. V. Matz, *Proc. Natl. Acad. Sci.* **2002**, *99*, 4256.

[9] H. Shinoda, Y. Ma, R. Nakashima, K. Sakurai, T. Matsuda, T. Nagai, *Cell Chem. Biol.* **2018**, *25*, 330.

[10] X. Shu, A. Royant, M. Z. Lin, T. A. Aguilera, V. Lev-Ram, P. A. Steinbach, R. Y. Tsien, *Science* **2009**, *324*, 804.

[11] J. Wiedenmann, S. Ivanchenko, F. Oswald, F. Schmitt, C. Röcker, A. Salih, K.-D. Spindler, G. U. Nienhaus, *Proc. Natl. Acad. Sci.* **2004**, *101*, 15905.

[12] E. M. Merzlyak, J. Goedhart, D. Shcherbo, M. E. Bulina, A. S. Shcheglov, A. F. Fradkov, A. Gaintzeva, K. A. Lukyanov, S. Lukyanov, T. W. J. Gadella, D. M. Chudakov, *Nat. Methods* **2007**, *4*, 555.

[13] J. Wiedenmann, A. Schenk, C. Röcker, A. Girod, K.-D. Spindler, G. U. Nienhaus, *Proc. Natl. Acad. Sci.* **2002**, *99*, 11646.

[14] N. G. Gurskaya, A. F. Fradkov, A. Terskikh, M. V. Matz, Y. A. Labas, V. I. Martynov, Y. G. Yanushevich, K. A. Lukyanov, S. A. Lukyanov, *FEBS Lett.* **2001**, *507*, 16.

[15] H. Tsutsui, S. Karasawa, H. Shimizu, N. Nukina, A. Miyawaki, *EMBO Rep.* **2005**, *6*, 233.

[16] S. Karasawa, T. Araki, T. Nagai, H. Mizuno, A. Miyawaki, *Biochem. J.* **2004**, *381*, 307.

[17] N. C. Shaner, G. G. Lambert, A. Chammas, Y. Ni, P. J. Cranfill, M. A. Baird, B. R. Sell, J. R. Allen, R. N. Day, M. Israelsson, M. W. Davidson, J. Wang, *Nat. Methods* **2013**, *10*, 407.

[18] T. Kogure, S. Karasawa, T. Araki, K. Saito, M. Kinjo, A. Miyawaki, *Nat. Biotechnol.* **2006**, *24*, 577.

[19] D. S. Bindels, L. Haarbosch, L. van Weeren, M. Postma, K. E. Wiese, M. Mastop, S. Aumonier, G. Gotthard, A. Royant, M. A. Hink, T. W. J. Gadella, *Nat. Methods* **2017**, *14*, 53.

[20] K. Katoh, J. Rozewicki, K. D. Yamada, *Briefings in Bioinformatics* **2019**, *20*, 1160.

[21] R. D. Finn, J. Clements, S. R. Eddy, *Nucleic Acids Res.* **2011**, *39*, W29.

[22] J. Banda-Vázquez, S. Shanmugaratnam, R. Rodríguez-Sotres, A. Torres-Larios, B. Höcker, A. Sosa-Peinado, *Protein Sci.* **2018**, *27*, 957.

[23] M. Slobodyanyuk, J. A. Banda-Vázquez, M. J. Thompson, R. A. Dean, J. E. Baenziger, R. A. Chica, C. J. B. daCosta, *Commun. Biol.* **2022**, *5*, 1264.

[24] W. Li, A. Godzik, *Bioinformatics* **2006**, *22*, 1658.

[25] W. Cai, J. Pei, N. V. Grishin, *BMC Evol. Biol.* **2004**, *4*, 33.

[26] F. Gabler, S.-Z. Nam, S. Till, M. Mirdita, M. Steinegger, J. Söding, A. N. Lupas, V. Alva, *Curr. Protoc. Bioinf.* **2020**, *72*, e108.

[27] L. Zimmermann, A. Stephens, S.-Z. Nam, D. Rau, J. Kübler, M. Lozajic, F. Gabler, J. Söding, A. N. Lupas, V. Alva, *J. Mol. Biol.* **2018**, *430*, 2237.

[28] H. Kim, T. Zou, C. Modi, K. Dörner, T. J. Grunkemeyer, L. Chen, R. Fromme, M. V. Matz, S. B. Ozkan, R. M. Wachter, *Structure* **2015**, *23*, 34.

[29] D. W. Close, C. D. Paul, P. S. Langan, M. C. J. Wilce, D. A. K. Traore, R. Halfmann, R. C. Rocha, G. S. Waldo, R. J. Payne, J. B. Rucker, M. Prescott, A. R. M. Bradbury, *Proteins:Struct., Funct., Bioinf.* **2015**, *83*, 1225.

[30] Q. Wang, L. J. Byrnes, B. Shui, U. F. Röhrig, A. Singh, D. M. Chudakov, S. Lukyanov, W. R. Zipfel, M. I. Kotlikoff, H. Sondermann, *PLoS One* **2011**, *6*, e23513.

[31] A. Sugizaki, K. Sato, K. Chiba, K. Saito, M. Kawagishi, Y. Tomabechi, S. B. Mehta, H. Ishii, N. Sakai, M. Shirouzu, T. Tani, S. Terada, *Proc. Natl. Acad. Sci.* **2021**, *118*, e2019071118.

[32] V. Z. Pletnev, N. V. Pletneva, K. S. Sarkisyan, A. S. Mishin, K. A. Lukyanov, E. A. Goryacheva, R. H. Ziganshin, Z. Dauter, S. Pletnev, *Acta Crystallogr., Sect. D:Biol. Crystallogr.* **2015**, *71*, 1699.

[33] D. J. Leibly, M. A. Arbing, I. Pashkov, N. DeVore, G. S. Waldo, T. C. Terwilliger, T. O. Yeates, *Structure* **2015**, *23*, 1754.

[34] M. Patrian, A. Shaukat, M. Nieddu, J. A. Banda-Vázquez, J. V. I. Timonen, J. P. Fuenzalida Werner, E. Anaya-Plaza, M. A. Kostiainen, R. D. Costa, *ACS Nano* **2023**,*17*(21), 21206.

[35] S. Grümbel, M. Hasler, S. Ferrara, M. Patrian, J. A. Banda-Vázquez, P. B. Coto, J. P. Fuenzalida Werner, R. D. Costa, *Adv. Opt. Mater.* **2024**, *12*, 2400106.

[36] M. D. Weber, L. Niklaus, M. Pröschel, P. B. Coto, U. Sonnewald, R. D. Costa, *Adv. Mater.* **2015**, *27*, 5493.

[37] X. Wang, Z. Li, W. Ying, D. Chen, P. Li, Z. Deng, X. Peng, *J. Mater. Chem. C* **2019**, *8*, 240.

[38] S. Sadeghi, R. Melikov, D. Conkar, E. N. Firat-Karalar and S. Nizamoglu, *Adv. Mater. Tech.* **2020**, *5*, 2000061.

[39] M. Nieddu, M. Patrian, S. Ferrara, J. P. Fuenzalida Werner, F. Kohler, E. Anaya-Plaza, M. A. Kostiainen, H. Dietz, J. R. Berenguer, R. D. Costa, *Adv. Sci.* **2023**, *10*, 2300069.

[40] B. Lim, J. Kim, M. S. Desai, W. Wu, I. Chae, S. W. Lee, *Biomacromolecules* **2023**, *24*, 118.

[41] D. Gutiérrez‐Armayor, Y.Atoini, D. Van Opdenbosch, C. Zollfrank, M. Nieddu, R. D. Costa, *Adv. Mater.* **2024**, *36*, 2311031.

[42] V. Fernández-Luna, D. Sánchez-de Alcázar, J. P. Fernández-Blázquez, A. L. Cortajarena, P. B. Coto, R. D. Costa, *Adv. Funct. Mater.* **2019**, *29*, 1904356.

[43] M. Patrian, M. Nieddu, J. A. Banda-Vázquez, D. Gutiérrez-Armayor, G. González-Gaitano, J. P. Fuenzalida-Werner, R. D. Costa, *Adv. Mater.* **2023**, *35*, 2303993

[44] M. Hasler, M. Patrian, J. A. Banda-Vázquez, S. Ferrara, A. C. Stiel, J. Fuenzalida-Werner, R. D. Costa, *Adv. Funct. Mater.* **2024**, *34*, 2301820.

[45] S. Ferrara, J. P. Fernandéz-Blázquez, J. P. Fuenzalida Werner, R. D. Costa, *Adv. Funct. Mater.* **2023**, *33*, 2300350.

[46] Y. Duan, C. Wu, S. Chowdhury, M. C. Lee, G. Xiong, W. Zhang, R. Yang, P. Cieplak, R. Luo, T. Lee, J. Caldwell, J. Wang, P. Kollman, *J. Comput. Chem.* **2003**, *24*, 1999.

[47] J. Wang, W. Wang, P. A. Kollman, D. A. Case, *J. Mol. Graphics* **2006**, *25*, 247.

[48] D. A. Case, K. Belfon, I. Y. Ben-Shalom, S. R. Brozell, D. S. Cerutti, T. E. Cheatham, III, V. W. D. Cruzeiro, T. A. Darden, R. E. Duke, G. Giambasu, M. K. Gilson, H. Gohlke, A. W. Goetz, R. Harris, S. Izadi, S. A. Izmailov, K. Kasavajhala, A. Kovalenko, R. Krasny, T. Kurtzman, T. S. Lee, S. LeGrand, P. Li, C. Lin, J. Liu, T. Luchko, R. Luo, V. Man, K. M. Merz, Y. Miao, O. Mikhailovskii, G. Monard, H. Nguyen, A. Onufriev, F. Pan, S. Pantano, R. Qi, D. R. Roe, A. Roitberg, C. Sagui, S. Schott-Verdugo, J. Shen, C. L. Simmerling, N. R. Skrynnikov, J. Smith, J. Swails, R. C. Walker, J. Wang, L. Wilson, R. M. Wolf, X. Wu, Y. Xiong, Y. Xue, D. M. York, P. A. Kollman, AMBER 2020, University of California, San Francisco. **2020.**

[49] J. Wang, R. M. Wolf, J. W. Caldwell, P. A. Kollman, D. A. Case, *J. Comput. Chem.* **2004**, *25*, 1157.

[50] K. Nienhaus, G. U. Nienhaus, J. Wiedenmann, H. Nar, *Proc. Natl. Acad. Sci.* **2005**, *102*, 9156.

[51] C. R. Søndergaard, M. H. M. Olsson, M. Rostkowski, J. H. Jensen, *J. Chem. Theory Comput.* **2011**, *7*, 2284.

[52] M. H. M. Olsson, C. R. Søndergaard, M. Rostkowski, J. H. Jensen, *J. Chem. Theory Comput.* **2011**, *7*, 525.

[53] W. L. Jorgensen, J. Chandrasekhar, J. D. Madura, R. W. Impey, M. L. Klein, *J. Chem. Phys.* **1983**, *79*, 926.

[54] J. C. Phillips, D. J. Hardy, J. D. C. Maia, J. E. Stone, J. V. Ribeiro, R. C. Bernardi, R. Buch, G. Fiorin, J. Hénin, W. Jiang, R. McGreevy, M. C. R. Melo, B. K. Radak, R. D. Skeel, A. Singharoy, Y. Wang, B. Roux, A. Aksimentiev, Z. Luthey-Schulten, L. V. Kalé, K. Schulten, C. Chipot, E. Tajkhorshid, *J. Chem. Phys.* **2020**, *153*, 044130.

[55] P. Eastman, R. Galvelis, R. P. Peláez, C. R. A. Abreu, S. E. Farr, E. Gallicchio, A. Gorenko, M. M. Henry, F. Hu, J. Huang, A. Krämer, J. Michel, J. A. Mitchell, V. S. Pande, J. P. Rodrigues, J. Rodriguez-Guerra, A. C. Simmonett, S. Singh, J. Swails, P. Turner, Y. Wang, I. Zhang, J. D. Chodera, G. De Fabritiis, T. E. Markland, *J. Phys. Chem. B* **2024**, *128*, 109.

[56] R. C. Walker, M. F. Crowley, D. A. Case, *J. Comput. Chem.* **2008**, *29*, 1019.

[57] G. de M. Seabra, R. C. Walker, M. Elstner, D. A. Case, A. E. Roitberg, *J. Phys. Chem. A* **2007**, *111*, 5655.

[58] M. Gaus, Q. Cui, M. Elstner, *J. Chem. Theory Comput.* **2011**, *7*, 931.

[59] M. Gaus, A. Goez, M. Elstner, *J. Chem. Theory Comput.* **2013**, *9*, 338.
